# Phage-specific metabolic reprogramming of virocells

**DOI:** 10.1038/s41396-019-0580-z

**Published:** 2020-01-02

**Authors:** Cristina Howard-Varona, Morgan M. Lindback, G. Eric Bastien, Natalie Solonenko, Ahmed A. Zayed, HoBin Jang, Bill Andreopoulos, Heather M. Brewer, Tijana Glavina del Rio, Joshua N. Adkins, Subhadeep Paul, Matthew B. Sullivan, Melissa B. Duhaime

**Affiliations:** 1 Department of Microbiology, The Ohio State University, 484 W 12th Ave, Columbus, OH 43210 USA; 2 Department of Ecology and Evolutionary Biology, University of Michigan, 1105 North University Ave, Ann Arbor, MI 48109 USA; 3 US Department of Energy Joint Genome Institute, 1800 Mitchell Dr #100, Walnut Creek, CA 94598 USA; 4 Environmental Molecular Sciences Laboratory, Pacific Northwest National Laboratory (PNNL), 902 Battelle Blvd, Richland, WA 99354 USA; 5 Biological Science Division, PNNL, 902 Battelle Blvd, Richland, WA 99354 USA; 6 Department of Statistics, The Ohio State University, 1958 Neil Ave, Columbus, OH 43210 USA; 7 Department of Civil, Environmental and Geodetic Engineering, The Ohio State University, 2070 Neil Ave, Columbus, OH 43210 USA; 8 Center for RNA Biology, The Ohio State University, 484 W. 12th Ave, Columbus, OH 43210 USA

**Keywords:** Bacteriophages, Microbial ecology

## Abstract

Ocean viruses are abundant and infect 20–40% of surface microbes. Infected cells, termed virocells, are thus a predominant microbial state. Yet, virocells and their ecosystem impacts are understudied, thus precluding their incorporation into ecosystem models. Here we investigated how unrelated bacterial viruses (phages) reprogram one host into contrasting virocells with different potential ecosystem footprints. We independently infected the marine *Pseudoalteromonas* bacterium with siphovirus PSA-HS2 and podovirus PSA-HP1. Time-resolved multi-omics unveiled drastically different metabolic reprogramming and resource requirements by each virocell, which were related to phage–host genomic complementarity and viral fitness. Namely, HS2 was more complementary to the host in nucleotides and amino acids, and fitter during infection than HP1. Functionally, HS2 virocells hardly differed from uninfected cells, with minimal host metabolism impacts. HS2 virocells repressed energy-consuming metabolisms, including motility and translation. Contrastingly, HP1 virocells substantially differed from uninfected cells. They repressed host transcription, responded to infection continuously, and drastically reprogrammed resource acquisition, central carbon and energy metabolisms. Ecologically, this work suggests that one cell, infected versus uninfected, can have immensely different metabolisms that affect the ecosystem differently. Finally, we relate phage–host genome complementarity, virocell metabolic reprogramming, and viral fitness in a conceptual model to guide incorporating viruses into ecosystem models.

## Introduction

Microbial metabolisms underlie ocean biochemistry [1], driving elemental fluxes and nutrient flow on a global scale. Viruses impact these processes through mortality, horizontal gene transfer, and the reprogramming of microbial energy-generating and matter-transforming metabolisms [2–4]. Because ~20–40% of surface ocean microbes are infected at any given time [2], infected cells—termed virocells [5–7]—are a major ecosystem feature. As virocells are metabolically and physiologically distinct from uninfected cells [8, 9], they occupy distinct ecological niches. Yet, little is known about how bacterial virocells are metabolically reprogrammed by their viruses (phages) to fuel phage energy and resource demands, or how this impacts cellular outputs and ecosystem functions. Determining and predicting the ecological implications of phage infections is a challenge in microbial ecology, but is the next step needed to better integrate phages into ecosystem models [10].

Metagenomics has enabled us to resolve population-level viral biodiversity globally [11–21], where “population” has been resolved from extensive genetics-grounded studies in cyanophages [22, 23], *Pseudoalteromonas* phages [24], reference-database phages [25], and from viromics-enabled studies [21]. Metagenomics answers *“who is there?”*. However, we cannot yet link this diversity to the consequences of infection, including addressing *“what are they doing?”* and *“how are they doing it?”*. This knowledge gap is closing with model-system-based, phage–host experiments where time-resolved genome-wide transcriptomics, proteomics, and metabolomics are unveiling infection characteristics in both environmental and medical systems [26–32]. These findings consistently demonstrate that phage gene expression has a predictable program whereby expressed genes fall into similar temporally regulated categories regardless of the host [26, 27, 30, 31]. However, host-specific responses to infection vary widely across phage–host pairs [27, 29–31, 33], and can modulate phage resistance. Such work shows that host resistance in nature derives not just from individual mutations or isolated mechanisms, such as mutations in phage receptors or host defenses against newly acquired phage DNA (e.g., restriction modification enzymes, CRISPRs, BREX, DISARM) [34]. Instead, there is an intracellular battle between phage and host, whereby a phage can be challenged simultaneously at multiple steps of the infection cycle, from adsorption through cell lysis [9, 30, 31, 33]. These new ‘omics approaches help to bridge the recognized gap between known sequences and unknown virocell biology [5, 9] by unveiling both the variety of strategies governing virocell responses and, in turn, what specific measurements could be done to quantify virocell ecosystem footprints. Thus, knowledge of the virocell at the molecular scale is foundational for understanding the biochemical impacts of viral infection, which together is critical knowledge for modeling the ecological role of virocells in nature [10].

Here we employ time resolved, genome-wide transcriptomics, and proteomics to better understand virocell metabolic reprogramming and ecology through *Pseudoalteromonas* phage–host interactions. The marine bacterium *Pseudoalteromonas* and its phages offer a tractable phage–host model system to study virocell ecology because genomic and life history traits of several *Pseudoalteromonas* hosts and phages have been described [35–39]. As marine *Pseudoalteromonas* spp. are among the most abundant particle-associated taxa in the ocean [40, 41] and the heterotrophic genus most predictive of carbon export from the surface to deep ocean [42], their phages likely impact global carbon cycling in undetermined ways. To better understand the role *Pseudoalteromonas* virocells may play in ecosystem processes, we followed infection of *Pseudoalteromonas* sp. str. 13–15 [24] (herein “host”) by a podophage (PSA-HP1, herein “HP1”) and a siphophage (PSA-HS2, herein “HS2”) [24] independently via time-resolved transcriptomics and proteomics. We assessed phage and host response to one another, and aggregated the findings into a new conceptual model as a baseline for quantifying potential ecosystem footprints and incorporating these processes into virus-explicit ecosystem models.

## Materials and methods

### Growth and infections

Growth and infection were conducted as described previously [24, 43–45]. Briefly, *Pseudoalteromonas* sp 13–15 cells were grown in an orbital shaker shaking at 150 rpm at 21 °C in 1%Z + CNP medium (26 g/L sea salts, 1 g/L yeast extract, 5 g/L proteose, 8.3 mM ammonium sulfate, 0.15 mM phosphoric acid, and 11 mM glucose added after autoclaving). A colony was grown overnight in 10 mL before 5 × 10^8^ cells were transferred to 200 mL in 1 L flasks and grown to mid-to-late-exponential phase. Then, 1 × 10^8^ cells were independently infected with PSA-HP1 or PSA-HS2 at a multiplicity of infection (MOI) of 0.1 for adsorption kinetics and initial one-step growth curves, or at MOI~5 for the time-resolved ‘omics experiments. Adsorption kinetics samples were taken immediately and every 5 min for 25 min. One-step growth curves samples were incubated for 15 min for phage adsorption, and then diluted 100-fold in 250 ml flasks for initial one-step growth curves, or tenfold in 1 L bottles for ‘omics sampling, as previously described [30, 31]. Cells were spread on Zobell plates and incubated for 2 days at room temperature (RT). Phages were enumerated via the top-agar plating technique [46]: cells were removed via 0.2-µm filtration, filtrate was serial diluted in artificial seawater (26 g/L sea salts) and mixed with 0.4 ml of bacterial overnight culture and 3.5 ml molten soft agar (0.6% low melting point agarose) before dispersing on agar plates, which were incubated 1–2 days at RT.

### Genome-wide transcriptomics

From diluted infections or controls, 25 ml were collected in biological triplicates and pelleted for 11 min at 20,000 g, and supernatant was discarded before flash-freezing in liquid N_2_. RNA was extracted using the Zymo Quick RNA Mini kit (R1054). RNA concentration and integrity were assessed using the Agilent 2100 Bioanalyzer RNA 6000 Pico assay with the prokaryote protocol. Ribo-Zero was used for removing rRNA and libraries were prepared with TruSeq Stranded Total RNA HT with total RNA starting material of 100 ng per sample and ten cycles of Polymerase Chain Reaction (PCR) for library amplification. Libraries were quantified using KAPA Biosystem’s next-generation sequencing library quantitative-PCR kit and run on a Roche LightCycler 480 real-time PCR instrument. The libraries were then multiplexed with other libraries and together prepared for sequencing on the Illumina HiSeq sequencing platform utilizing a TruSeq paired-end cluster kit, v4, and Illumina’s cBot instrument to generate a clustered flow cell for sequencing. Glow cell sequencing was performed on the Illumina HiSeq2500 sequencer using HiSeq TruSeq SBS sequencing kits, v4, following a 2 × 100 indexed run recipe.

Sequencing depth per sample ranged from 0.1 to 24 million reads (mean = 16.9 million reads, median = 16.7 million reads) (Supplementary Dataset, Table 3). Raw reads were filtered with the default JGI pipeline using BBtools v36.21 to remove all reads containing ≥ 2 “N” bases, an average read quality score of <10, read length <49 bp, containing known Illumina artifacts, or mapping to PhiX, human, cat, dog and mouse genomes with ≥93% identity. Reads were trimmed to remove known Illumina artifacts in 5′ and 3′ ends, and when the 3′ base quality score was <6 on 3′. Filtered reads were mapped to *Pseudoalteromonas* sp. strain 13–15 and phages PSA-HP1 and PSA-HS2 (downloaded from GenBank on 11 January 2017, 6 December 2014, and 21 November 2014, respectively) (Supplementary Fig. S1), using BBmap v36.84 (options ambiguous = toss), a threshold of 90% nucleotide identity, and maximum insertion/deletion size = 4. FeatureCounts counted the number of fragments (read pairs) that mapped completely within each gene. FeatureCounts also computed read strandedness (i.e., whether reads were originated from the reverse strand instead of from both): reads mapping to the reverse strand divided by those mapping to both strands. The threshold was >95%, which ensures robust comparison across genes and libraries.

Read normalization and differential expression analyses were performed following previous studies [30, 31]. Briefly, host and phage reads (Supplementary Dataset, Tables 4–6) were normalized separately by dividing the number of reads by the gene length and sequencing depth (i.e., FPKM [47]). Phage genes were clustered by temporal expression profile (from *z*-score transformed log2FPKM values) using Pearson’s correlation and resampling using the clusterStab R package [48] as previously described [49]. Temporal clusters were manually adjusted through plotting. Differential expression (DE) was calculated between infected and uninfected samples by time point using edgeR [50]. Genes with *p* values < 0.01 and false discovery rate < 0.05 were considered DE. Fold change (log_2_FC) was calculated as the expression difference between infected and control, after accounting for fraction of infected cells (Supplementary Dataset, Table 7). Overexpression is when log_2_FC > 0 and underexpression when log_2_FC < 0. Heatmaps were generated with the R package *pheatmap* (http://CRAN.R-project.org/package = pheatmap).

### Genome-wide proteomics

From diluted infections or controls, 80 mL were collected in biological triplicates and pelleted for 8 min at 20,000 g. The supernatant was discarded prior to flash-freezing with liquid N_2_. Proteomes had low quantity and required filter-enrichment (see Supplementary Methods). A Waters nano-Acquity M-Class dual pumping UPLC system (Milford, MA) was configured for on-line trapping of a 5 µL injection at 3 µL/min with reverse-flow elution onto the analytical column at 300 nL/min. Columns were packed in-house using 360 µm o.d. fused silica (Polymicro Technologies Inc., Phoenix, AZ) with 5-mm sol–gel frits for medium retention and contained Jupiter C18 medium (Phenomenex, Torrence, CA) in 5 µm particle size for the trapping column (150 µm i.d. × 4 cm long) and 3 µm particle size for the analytical column (75 µm i.d. × 70 cm long). Mobile phases consisted of (A) 0.1% formic acid in water and (B) 0.1% formic acid in acetonitrile with the following gradient profile (min, %B): 0, 1; 2, 8; 20, 12; 75, 30; 97, 45; 100, 95; 110, 95; 115, 1; 150, 1.

Mass spectrometry (MS) analysis was performed using a Q-Exactive Plus mass spectrometer (Thermo Scientific, San Jose, CA) outfitted with a home-made nano-electrospray ionization interface. Electrospray emitters were prepared using 150 µm o.d. × 20 um i.d. chemically etched fused silica. The ion transfer tube temperature and spray voltage were 300 °C and 2.2 kV, respectively. Data were collected for 100 min following a 15 min delay from sample injection. FT-MS spectra were acquired from 300 to 1800 m/z at a resolution of 35k (AGC target 3 × 10^6^) and while the top 12 FT-HCD-MS/MS spectra were acquired in data dependent mode with an isolation window of 2.0 m/z and at a resolution of 17.5k (AGC target 1 × 10^5^) using a normalized collision energy of 30 and a 30 s exclusion time. Raw protein counts were used to evaluate the quality of the biological replicates (see Supplementary Methods and Supplementary Figs. S11–S13). Downstream analyses were done as previously [31] whereby counts were *z*-score transformed for all host samples or each phage, and represented in heatmaps (see above).

### Global transcriptome and proteome

ANOVA analyses of linear models that attempt to predict the counts with treatment type (uninfected, HP1 infected, HS2 infected), and a linear and quadratic function of time and interaction between infection type and time, were applied to the raw transcriptomics and proteomics counts. The fitted curves of transcript and protein counts and 95% Bonferroni corrected confidence intervals. Pairwise comparisons compared uninfected hosts, HP1 infected, and HS2 infected hosts to each other in overall counts and linear time trend using Tukey’s method controlling for multiple comparisons.

### Phage–host complementarity

Codon counts, codon relative frequencies, and Relative Synonymous Codon Usage (RSCU) values were calculated using the “uco” function from the R *seqinr* package. Cosine distance measured the distance between the vectors of host and each phages’ codon frequencies such that *D*_*c*_*(phage, host)* = 0 for two genomes with identical codon frequencies. To determine the codons (and consequently, amino acids) with largest effect on cosine distance, each phage-vector’s codon frequency was iteratively set to the host frequency and *D*_*c*_*(phage, host)* re-measured. The difference between actual and iteratively-derived cosine distances (abs(*D*_*c*_*(phage, host)’*  *−* *D*_*c*_*(phage, host))* represented each codon’s impact on the overall phage–host codon mismatch (i.e., “codon impact”). To determine whether HP1-host codon mismatch was significantly different from other phage–host pairs, a pairwise independent *t*-test with Holm–Bonferroni adjusted *p* values for multiple hypotheses testing was performed with 1185 phage–host pairs that had complete genomes (downloaded from RefSeq on 10 June 2019). Additional details are provided in Supplementary information.

## Results and discussion

### Both phages infect efficiently, but with different host-takeover strategies

**Fig. 1:**
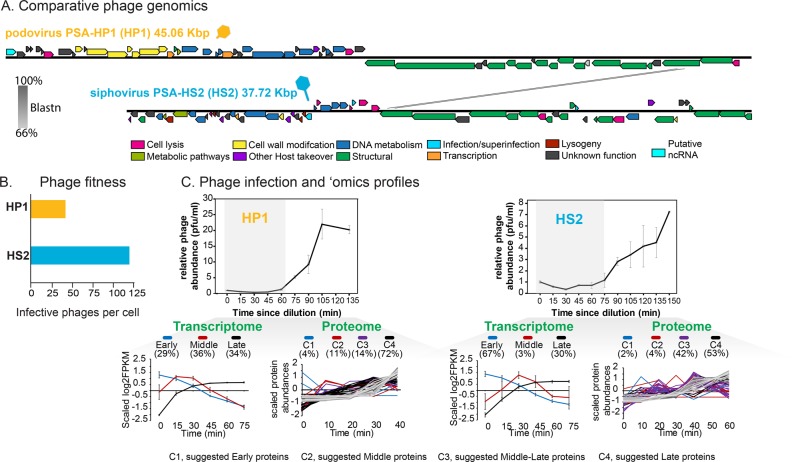
Podophages PSA-HP1 (HP1) and PSA-HS2 (HS2). **a** Blastn-based phage genome comparison. **b** Phage fitness on *Pseudoalteromonas* str. 13–15, defined as number of infective phages produced per cell. **c** Temporal dynamics of phage infection (0 = 15 min after phage addition) to measure latent periods and ‘omics profiles. *Transcriptome*: average and standard deviation of scaled gene expression classified as early (blue), middle (red), or late (black). *Proteome*: detected proteins and their scaled abundances, colored following the transcript clusters. Parentheses contain either the fraction of total genes expressed or of proteins detected. Pfu particle forming units.

Based on gene sharing networks, phage HP1 is related to the T7 podophage group (Supplementary Fig. S2). Phage HS2 is a siphovirus and putative temperate phage, given its multiple predicted lysogeny genes [24]. The phages share only 1% of their genomes (Fig. 1a and Supplementary Fig. S2), and HS2 has threefold higher fitness (when fitness is infective particles produced per burst) than HP1 on the host used here (Fig. 1b [24]). The mechanisms of infection of these phages remain uncharacterized. We assessed adsorption kinetics and infection dynamics, which revealed that, for both phages, 80% of virions adsorbed within 5 min, and their latent periods lasted 60 min (Fig.1c and Supplementary Fig. S3). Transcriptomic sequencing revealed that transcriptomes of both phages were organized into three temporal stages (*early, middle*, and *late;* Fig. 1c and Supplementary Figs. S4 and S5), with proteins appearing shortly thereafter (Fig. 1c and Supplementary Figs. S6 and S7). The similar and relatively rapid adsorption kinetics and latent periods, as well as the close coupling of the transcriptome and proteome suggest that both phage infections are efficient on this host, as described in other time-resolved marine phage–host studies [27, 30, 31].

For both phages, the structural genes were transcribed late (Supplementary Fig. S5d, g; Supplementary Tables S1 and S2), as commonly observed [51], but HS2’s relative protein abundances of the late transcribed and predicted structural genes were higher than those of HP1 (Supplementary Fig. S8). Transcription of the early and middle genes, however, differed between the phages. Specifically, 29 and 36% of HP1 genes were expressed early and middle, respectively (Fig. 1c and Supplementary Table S1), whereas 67 and 3% of HS2 genes were expressed early and middle, respectively (Fig. 1c and Supplementary Table S2). Further, their host takeover capacity differed. The predicted peptidoglycan modification genes encoded by HP1 [24] were expressed immediately, followed by its host takeover genes (e.g., MazG, σ-70 transcriptional factor) and DNA metabolism genes (e.g., helicases, nucleases, and T7-like DNA polymerases (DNAP)) known to degrade host DNA, recycle nucleotides, and replicate phage DNA [51] (Supplementary Fig. S5a–c and Supplementary Table S1). In contrast, HS2 expressed DNA metabolism genes early (e.g., DNA helicase, DNA recombination, and repair; Supplementary Fig. S5a and Supplementary Table S2), but it lacks the large group of host interaction and takeover genes and the DNAP encoded by HP1 (Fig. 1a and Supplementary Tables S1 and S2).

Together these phage-focused findings reveal that unrelated phages can infect the same host efficiently, but with different host-takeover strategies and different fitnesses.

### Global patterns of host takeover within the contrasting virocells

**Fig. 2:**
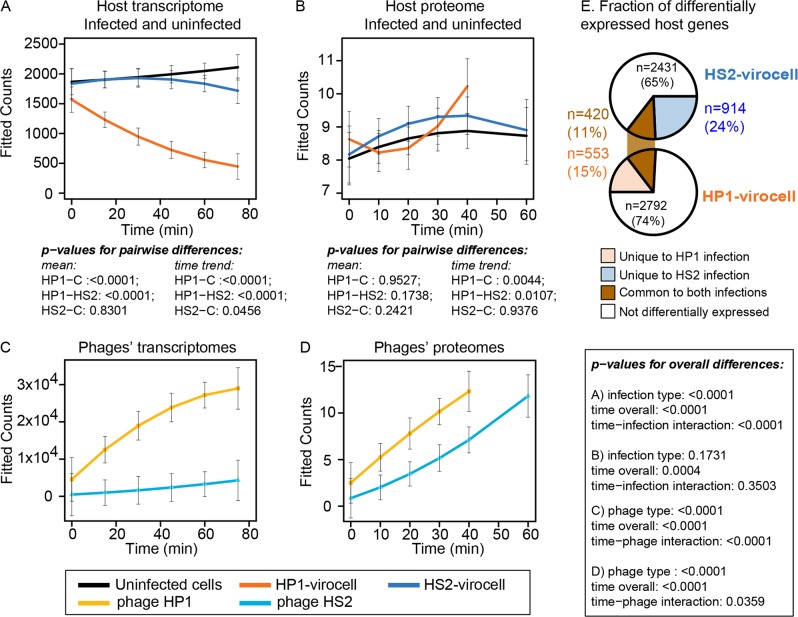
Host global transcriptome and proteome takeover by phage. **a** Temporal fitted raw transcript counts for uninfected controls, HP1 infected (HP1 virocell), and HS2-infected (HS2 virocell) cells. **b** Temporal fitted protein counts for uninfected controls, HP1 virocells, and HS2 virocells. Temporal fitted raw transcript (**c**) or protein (**d**) HP1 and HS2 counts. For all, *p* values indicate confidence from the ANOVA analysis of a linear model predicting the counts with sample type (uninfected, HP1 infected, or HS2-infected cells), a linear and quadratic function of time, interaction between infection type and time, and the between-sample pairwise comparisons. The error bars indicate 95% Bonferroni-corrected simultaneous confidence intervals for the fitted response. All pairwise comparisons are multiple-comparison corrected using Tukey’s method. Time (min) = 0 indicates 15 min after diluting the infection. **e** Host genes differentially expressed with and without each phage.

With HP1 expressing more host interaction and takeover genes, we hypothesized that HP1-infected cells (i.e., HP1 virocells) would be more rapidly and significantly impacted than HS2-infected cells (i.e., HS2 virocells) (Supplementary Table S3). Overall, global levels of host transcription through time indicated that treatment type (uninfected, HP1 infected, HS2 infected) significantly impacted host transcript abundances (F-stat: 237.94, *p* value < 0.0001; Fig. 2a). While host transcript abundances continued increasing in uninfected cells, they decreased in both virocells and at a higher rate during HP1 infection (Fig. 2a). Specifically, the transcript counts in the HP1 virocell and the HS2 virocell decreased at 5.4× and 1.6× the rate of uninfected cells, respectively. These findings are consistent with the hypothesis that HP1, with its expressed host-takeover genes, would more rapidly and significantly impact host transcription than HS2.

Unlike transcription, however, overall virocell protein abundances were not significantly different across treatment types (F-stat: 1.75, *p* value: 0.173; Fig. 2b). Notably, bacterial transcripts and proteins are largely uncorrelated [52], protein turnover is slower than that of most transcripts [53], and proteins detected may derive from recycling old proteins or new production [53]. These findings highlight the need to complement global proteomics with transcriptomics to assess the rapidly changing elements of infection, such as phage response. The temporal analyses indicated that the host proteins in the HP1 virocell did increase at a higher rate than in uninfected cells and the HS2 virocell (*p* value < 0.01; mean difference: 0.056 and 0.052 protein counts per minute, respectively). This suggests that (i) the phages have a more notable impact on the levels of host transcripts than proteins, (ii) and that HP1 has a greater impact on both transcription and protein dynamics than HS2 (Fig. 2b).

After examining the host impacts, we next evaluated phage transcription and translation in each virocell (Supplementary Table S3). Significant differences in transcription levels were identified between all three variables tested: phage type (F-stat: 156.6, *p* value < 0.0001), time (F-stat: 44.82, *p* value < 0.0001), and the interaction between phage type and time (F-stat: 24.78, *p* value < 0.0001). This suggested that HP1’s genes were transcribed significantly (*p* value < 0.0001) more and faster (mean difference: 19880 transcript counts and 264 transcript counts per minute, respectively) than HS2’s genes (Fig. 2c). Protein translation also significantly differed across variables tested (phage type, *p* value < 0.0001; time, *p* value < 0.0001; and the interaction of variables, *p* value < 0.05). The overall trend in translation followed that of transcription: there were greater abundances of HP1 proteins (mean difference: 4.69 protein counts, F-stat: 19.98, *p* value < 0.0001) and these abundances increased faster in the HP1 virocell (F-stat: 4.40, *p* value: 0.0359) than for HS2 proteins in the HS2 virocell (Fig. 2d).

**Fig. 3:**
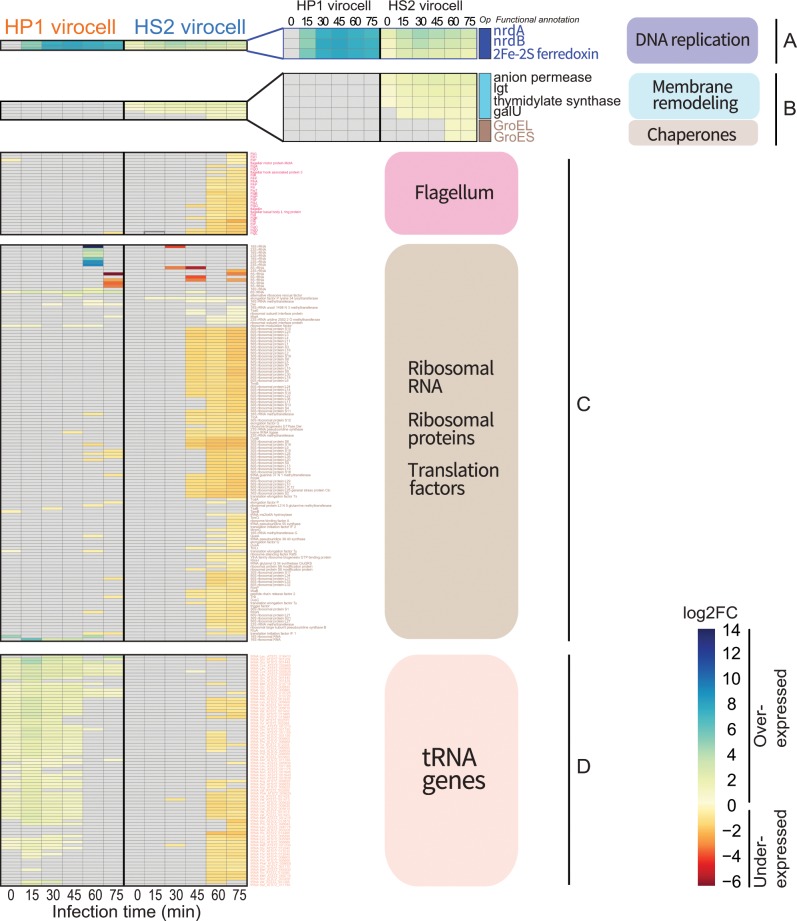
Host genes differentially expressed in both virocells. Heatmap representing select host genes’ fold change (log_2_FC) expression in infected vs uninfected, separated into categories: **a** The operon (“op”) containing the genes *nrdA*, *nrdB* and ferredoxin is the highest expressed group in both virocells. **b** The operon containing 5 genes mainly involved in cellular transport that comprises the group of most under-expressed genes in both virocells. **c** An operon with putative membrane remodeling genes and the chaperones GroEL/ES is the highest expressed group in the HS2 virocells. **d** Underexpression of both flagellar synthesis and assembly and protein translation genes (including ribosomal RNA, ribosomal proteins and translation factors) in the HS2 virocells. **e** Overexpression of tRNA genes in the HP1 virocells, which are under-expressed in the HS2 virocells.

Finally, we evaluated the common ways in which both virocells responded to infection by these unrelated phages. While half (*n* = 1887, 50%) of the host genes were differentially expressed (DE) in infected relative to uninfected cells, only 420 genes (11% of total genes) were commonly DE in both virocells (Fig. 2e; Dataset Table 7). Among those, here we focused on genes that were exceptionally over- and under-expressed. Only three genes were overexpressed ≥ 2-fold throughout the entire infection in both virocells: class I ribonucleoside diphosphate reductase subunits A (*nrdA*; temporal fold change ranging from 4 to 205) and B (*nrdB*; temporal fold change ranging from 2 to 239), and a 2Fe-2S-like ferredoxin (temporal fold change ranging from 2 to 220) (Fig. 3a). All three genes belong to the same host operon. The Nrd proteins catalyze the rate-limiting step of DNA synthesis by reducing ribonucleosides to ribonucleotides [54]. In *E. coli*, the adjacent ferredoxin is required for correct Nrd functioning by maintaining the cofactor associated with the *nrdA* and *nrdB* subunits [55]. Though Nrd genes are found across a wide range of coliphage [56] and environmental [57] phage genomes, neither HP1 nor HS2 encode *nrd* genes. Presumably these viruses repurpose host Nrd activity to replicate phage DNA.

Conversely, only five consecutive genes were underexpressed twofold or less in the infected cells, relative to the uninfected, throughout the infection in both virocells. These genes are efflux transporters involved in detoxification (e.g., of metals, antibiotics) and were underexpressed from twofold to 18-fold (Fig. 3b). Transcript underexpression may lead to diminished or abolished protein function of these efflux transporters in both virocells. Such under-expression of transporters contrasts with *C. jejuni* virocells where a multi-drug transporter was over-expressed during myovirus infection in this pathogen [58]. Future work is needed to experimentally evaluate the purpose of such phage-specific virocell responses to infection and their potential ties to pathogenesis.

Together these findings suggest that (i) virocell metabolic takeover impacts host gene transcription more than translation, (ii) host gene transcription in the HP1 virocell is shut-down faster and to a greater degree than in the HS2 virocell, and (iii) both virocells are reprogrammed to support phage DNA replication, as well as to shut down the function of specific efflux transporters. Such repression of host transcription may leave the host’s transcriptional machinery more available to be redirected towards increasing the phage transcripts in early infection, as was observed in the HP1 virocell. The greater takeover and reprogramming observed by the HP1 virocell to transcribe genes and increase phage proteins faster and to higher levels than in the HS2 virocell likely incurs a cost. Such a cost may partly explain its lower fitness relative to HS2, when measured under identical host and culture (i.e., environmental) conditions (Fig. 1b) [24]. Additional studies will be required to assess whether such an inverse relationship between degree of host takeover and phage fitness is universal across phage–host systems.

### The HS2-virocell conserves energy, while the HP1-virocell alters central carbon and energy metabolisms

We next evaluated phage-specific impacts on host gene expression. Of the total host genes DE within the virocells, 15% (*n* = 553) of those were unique to HP1 and 24% (*n* = 914) unique to HS2 (Fig. 2e; Dataset Table 7). During infection by the more fit HS2, 65% of host DE genes were expressed during *late* infection ( ≥ 45 min; Fig. 3; Dataset Table 7). This pattern contrasts with the more evenly distributed transcriptional response found in many other phage–host systems [28, 31, 32, 58–60]; though none of these studies involved siphoviruses. Whether this late transcriptomic response is a feature of HS2 or of siphoviruses in general—especially those suspected to integrate—is an open question, but it suggests that HS2 can largely utilize *existing* host resources to replicate itself.

The examination of overexpressed host genes in the HS2 virocell relative to uninfected cells revealed that the host responses were enhanced by phage infection. Here, genes with the highest expression throughout the infection (~2-fold to ~5-fold) included an operon with *tauE*-superfamily (anion permease), *lgt* (peptidoglycan modification), *thyA* (nucleotide metabolism), and *galU* (cell envelope synthesis) (Fig. 3c). ThyA is involved in the same pathway as the *nrd* genes, thus providing additional evidence for host reprogramming to support phage nucleotide synthesis. The rest of the expression patterns may reflect cellular surface changes to recycle membrane components or import extracellular compounds, as previously observed [61]. Two other over-expressed host genes were the adjacent chaperones *groEL/ES* (Fig. 3c), which in coliphage lambda assist virion head assembly [62]. These expression patterns suggest that remodeling the cell surface and leveraging the host’s machinery for phage DNA replication and virion assembly are among the greatest changes in the HS2 virocell.

Finally, for the HS2 virocell, the two largest categories of *underexpressed* genes included motility (27 flagellar synthesis and assembly genes) and translation (140 genes, including myriad 30 S and 50 S ribosomal proteins, translation factors, and tRNA genes), with fold-change values ranging from 1.3 to 49-fold under-expressed in the infected compared to uninfected cells (Fig. 3d; Dataset Table 7). While motility increased in a temperate marine phage–host system [39] it was selected against during phage infection in low temperature marine environments for energy conservation [63]. In addition, translation is the most energy-demanding process of viral replication [64]. Thus, together the underexpression of the high energy-demanding processes motility and translation in the HS2 virocell may represent an energy-conserving strategy during late infection.

Contrasting the HS2 virocell, no genes had sustained under-expression throughout the infection cycle in the HP1 virocell (Dataset Table 7). Instead, the HP1 virocell showed sustained gene over-expression, including a ligand-gated channel of the *tonB* superfamily (temporal fold-change of expression ranging from 2 to 231), and its neighboring ferredoxin (temporal fold-change of expression ranging from 3 to 875) (Dataset Table 7). As in other bacteria, these responses may lead to scavenging iron from the environment [65], which is pivotal in marine systems given that low oceanic iron concentrations limits microbial growth [66–68]. Future measurements of virocell-mediated iron scavenging may provide specific quantification to virocell-mediated ecosystem footprints. In addition, the other most highly over-expressed genes throughout the infection were 82 translation genes, including 39 ribosomal proteins and translation factors, and 43 tRNA genes, with up to 14-fold expression in infected compared to uninfected cells (Fig. 3d, e, Dataset Table 7). Phages often encode tRNA genes, which is thought to facilitate utilization and redirection of the host’s translational machinery towards making phage proteins across phages [69, 70]. However, as both HP1 and HS2 genomes lack tRNA genes, HP1 likely recruits host tRNAs for translating phage proteins instead of utilizing the ongoing host translational machinery, as HS2 presumably does. We hypothesized that this pattern stemmed from a greater mismatch between the host’s translational environment and HP1’s reproduction demands, relative to that of HS2.

**Fig. 4:**
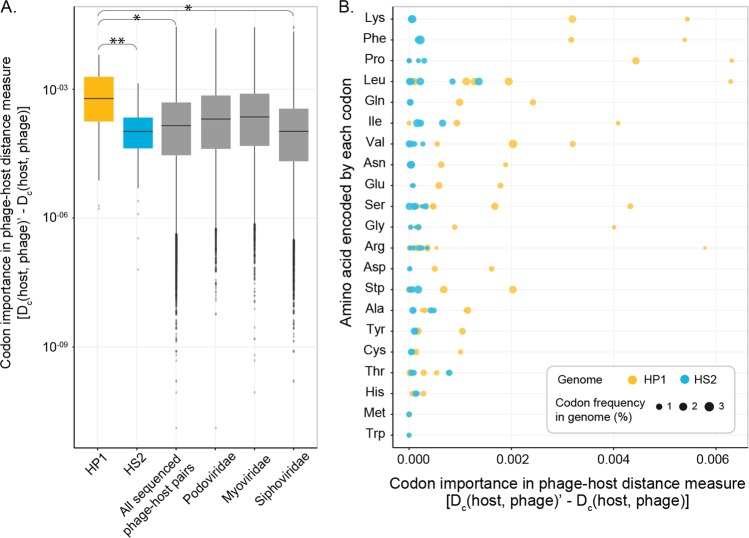
Dissimilarity between phage and host codons and amino acids. **a** Distribution of all codon importance measures for phage–host distances. Datasets include: HP1 or HS2 against *Pseudoalteromonas* str. 13–15, all sequenced phage–host pairs in RefSeq (*n* = 1187), and either the myoviridae (*n* = 229), podoviridae (*n* = 166), or siphoviridae (*n* = 671) phage–host pair subset. Greater values represent codons causing greater distances between phage and host codon frequency vectors. Box represents the interquartile range (IQR) with the middle line as the median. Whiskers extend to 1.5*IQR and dots are outlier values beyond that. Pairwise comparisons between all x-variables are significant (pairwise Wilcoxon test, *p* value < 0.05; Tables S4 and S5). Asterisks denote the HP1 significant comparisons described. **b** Each point represents the codon importance in the HP1 host and HS2-host similarity measures (*x*-axis, as in **a**). Synonymous codons are aggregated by the encoded amino acids (y-axis). The point size denotes phage genome codon frequency.

To test this hypothesis, we analyzed the %GC, codon, and amino acid complementarity between phages and host. The %GC was more different from host (39.8%) for HP1 (44.7%) than HS2 (40.2%). The degree of phage–host codon dissimilarity was greater for HP1 (codon distance, *D*_*c*_(HP1,host) = 0.088) than for HS2 (*D*_*c*_(HS2,host) = 0.011) (Supplementary Tables S4 and S5). Correspondingly, HP1 had significantly greater divergence from host codon biases than HS2 (*p* value = 0.0002; Fig. 4a). To contextualize this dissimilarity between HP1 and host codon biases, we calculated the average codon distance between all publicly available phage–host pairs. The HP1-host codon biases were significantly greater than all phage–host pairs (*p* value = 0.0330; Fig. 4). While the HP1-host codon biases were not significantly greater than the mean of the podoviral subset (*p* value = 0.1036), they were greater than the mean of the siphoviral subset (*p* value = 0.0075), which suggests a family-specific difference in phage–host codon biases (Fig. 4a; Supplementary Fig. S9). Such a trend has been observed in kmer frequency analyses of phages and their hosts [71], but this is the first exploration of the potential implications of phage–host genome complementarity on viral replication. Namely, relative to HS2, the greater HP1-host codon dissimilarity results in a greater mismatch between HP1 and host amino acids (Fig. 4b). As such, HP1 has a greater translational demand to synthesize proteins than HS2 on the same host.

**Fig. 5:**
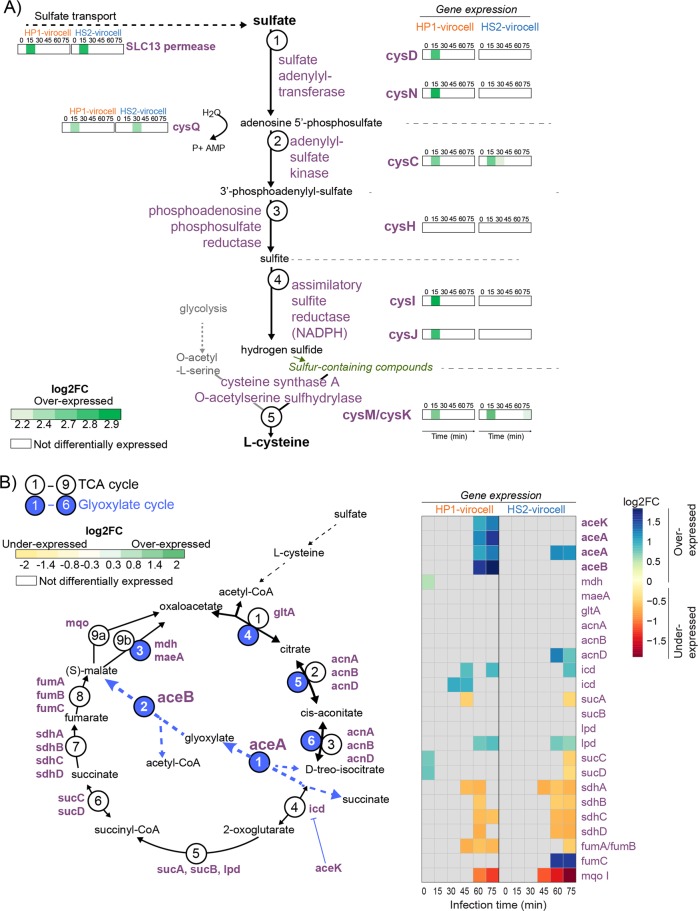
Phage-specific energy metabolism rewiring in virocells. **a** Sulfate intracellular transport and reduction to hydrogen sulfide, for cysteine production. Enzyme gene expression is shown as log2-fold change (log2FC; comparing infected vs uninfected). **b** The TCA cycle (black) with its glyoxylate bypass (blue), for the presumed consumption of cysteine. Each enzyme and its expression (log2FC in infected vs uninfected cells) is shown on the heatmap. For both, protein dynamics are represented in Fig. S7. For all expression, absence of virocell differential expression has white/gray background.

Given that translation is the most energetically costly phase of building viruses [64], we next sought to identify mechanisms by which HP1 may manage the heavier energetic burden of protein translation in this host. Among the highest expressed genes in the HP1 virocell were those involved in the assimilatory sulfate reduction pathway (Fig. 5a, Supplementary Fig. S10a). This pathway converts sulfate to hydrogen sulfide for incorporation into sulfur-containing compounds, most frequently the amino acid cysteine [72]. Given that HP1’s proteins are not substantially more enriched in cysteine than those of HS2 (Fig. 4b), we posit that cysteine synthesis in the HP1 virocell serves for energy production through C_2_ compounds instead of for translation. Specifically, cysteine can be degraded into acetyl-CoA that is then converted into energy, most commonly through the tricarboxylic acid (TCA) cycle [73]. Given that the TCA cycle is an energy-generating metabolism, we next investigated its expression in HP1 virocells to inquire whether it could be a primary energy source.

The most highly expressed genes (~3-fold) of the TCA cycle were those involved in the glyoxylate shunt: *aceA* and *aceB* (Fig. 5b, Supplementary Fig. S10b). The majority of the TCA cycle genes were either not DE or were differentially underexpressed (Fig. 5b). These findings suggest that the glyoxylate shunt was being used to increase cellular ATP and reducing power similarly to other bacteria grown on C_2_ compounds [74]. The glyoxylate shunt of the TCA cycle is commonly observed under various stressors [75], but has not been described during phage infection. Additionally, HP1 encodes a glutaredoxin gene (Supplementary Table S1), an iron-sulfur cluster assembly protein also found in other marine viruses [76], which is needed to make prosthetic groups for proteins in nucleotide synthesis, sulfur reduction, and the TCA and glyoxylate cycles [77]. This HP1 glutaredoxin gene, but none of the host-encoded glutaredoxin genes, was highly expressed at the same time as sulfur reduction and glyoxylate cycle genes and may have supported the redirection of metabolic flux through the glyoxylate shunt of the TCA cycle (Supplementary Fig. S5c and Dataset Table 7).

Together, these and the global host-takeover observations (Fig. 2) suggest that (i) the HS2 virocell generated an environment for phage reproduction whereby the phage used existing host metabolic resources while shutting down energy-costly processes no longer necessary for the phage towards the late stages of infection, and that (ii) HP1 virocells were reprogrammed to enhance translation and shuttle energy metabolisms by synthesizing sulfur-rich amino acids and degrading them for energy via the glyoxylate-TCA cycle. Given that HS2 has higher host complementarity and fitness than HP1, HS2 virocell may provide the intracellular environment and resources needed for infection success, whereas HP1 virocell greatly reprograms metabolisms to meet the demands of HP1 infection.

### A conceptual model for integrating virocells into viral ecology

Most of viral ecology—the study of interactions between viruses, organisms, and the environment—has focused on free viruses (the extracellular infectious stage of a virus), largely due to methodological limitations. Data describing free viruses, such as their abundances (including production rates [78]), virus-microbe ratios [79], diversity, and population-level biogeography via (meta)genome sequencing [11, 12, 21, 13–20], provides a rich context to infer *potential* interactions between viruses, other organisms [80–83], and the environment [3]. From an ecosystem perspective, the impact of this potential is realized via the reprogrammed metabolism of infected cells prior to lysis, and manifests as virocell-environment interactions. Globally, virocell metabolism has the potential to contribute just as much (and possibly more [84]) to ecosystem processes as the metabolism of uninfected cells in aquatic habitats [85]. Given the different resource requirements, metabolic transformations, and nutrient transport between a cell and a cell-turned-virus-factory [86, 87], virocells have a unique metabolic program that influences nutrient fluxes in microbial food webs [9] and merits their study to the depths we now understand their free viral counterparts.

**Fig. 6:**
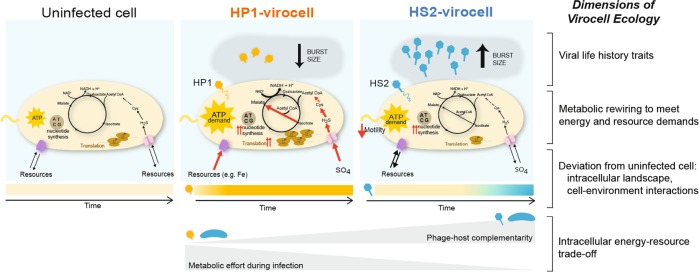
The dimensions of virocell ecology. Viral life history traits, e.g., burst size, adsorption and infection efficiency, latent period, impact viral fitness. The multi-omics analyses here have enabled the identification of mechanisms underlying these fitness-defining traits. During infection, the virocell undergoes *metabolic rewiring to meet energy and resource demands*. The greater *metabolic effort during infection* incurred by the HP1 virocell was evidenced by (i) an immediate, sustained, and more drastic *deviation from uninfected cell*, as seen in host transcription and protein levels; (ii) fast phage transcription and high accumulation of phage proteins; and (iii) rewiring host central carbon and energy metabolisms to meet the cost of creating more transcripts and proteins. In contrast, little work was invested by the HS2 virocell until past the midpoint of infection. This intracellular impact determines the degree to which the virocell *deviates from the uninfected cell* through time and, consequently, the environmental footprint of the virocell. We propose that a major determinant of the intracellular battle waged during infection is the phage–host complementarity of biomolecules (nucleotides, amino acids), which underlies an *intracellular energy-resource trade-off*. Namely, the phage with the highest degree of host complementarity (here, HS2) is able to access and utilize the available resources with minimal energetic effort, while minimizing the intracellular impact on the host and maximizing its fitness.

While pioneering studies have characterized altered virocell metabolisms [9, 33, 88], and how such metabolisms shape infection strategies and outcomes [9], much progress is needed to model the ecosystem impacts of phage infection. Our work seeks to push a foundational synthesis in viral ecology [10] to more explicitly include virocells by proposing a conceptual model that relates phage–host genomic complementarity, virocell metabolism, virocell energy-resource trade-offs, and virocell-environment interactions (Fig. 6). Specifically, our experimental design enabled us to follow two different infection trajectories that represent the outcome of trade-offs for infection resources. By examining two infections on one host under identical controlled laboratory conditions, both phages initially had access to the same resources for their replication. However, the manner and efficiency by which they accessed those resources—partly determined by phage–host biomolecule complementarity—varied. Specifically, HP1 was least complementary to the host in nucleotide, amino acid and codon composition, and thus had fewer intracellular resources (nucleotides and amino acids) available for recycling than did HS2. We propose that this lower complementarity led to higher metabolic demand in the HP1 virocell and, consequently, contributed to HP1’s lower relative fitness (smaller burst size), given that fitness is partially determined by a phage’s ability to access and leverage resources to infect [86, 87]. Because of the higher metabolic demand, HP1 virocells needed to more drastically augment translation and acquire resources, including scavenging iron and transporting sulfate into the cell to reduce it to cysteine for consumption via the glyoxylate cycle. Thus, the HP1 infection likely differentially impacted the extracellular-to-intracellular resource fluxes and the virocell microenvironment (Fig. 6). Our multi-omics-inferred observations provide a roadmap for future work to measure specific nutrient and metabolite dynamics (e.g., uptake, release) to better quantify virocell-derived ecosystem footprints. In summary, we propose phage–host genome complementarity as an important driver of the nature and magnitude of virocell reprogramming and a property that links virocell metabolism with infection outcomes (here, fitness defined by burst size). As such, different virulent phages are likely to uniquely impact biogeochemical transformations and virocell–cell interactions in their local microenvironment, which may consequently lead to phage-specific ecosystem footprints, even on the same host. These findings also lay the foundation for working towards identifying virocell “functional guilds” akin to those defined for macro-organisms [89]. We expect that as data grow, phage–host combinations that exploit resources in similar ways will emerge, regardless of taxonomic affiliation of phage or host. Simplifying community diversity by such “functional guilds” would allow incorporation of virocells into ecosystem models, just as other such simplifications have enabled models of global-scale ocean biogeochemistry [90] and microbial community assembly [91, 92].

## Conclusions

A common vision of lytic phage infection is one of a phage hijacking its host, commandeering its functions, and turning it into a phage reproduction machine until lysis [51]. Though phages are commonly now perceived as efficient masters of their microbial hosts [3], the nuances of host responses are likely many and varied with respect to interaction and takeover strategies [51, 62], including direct AMG-driven metabolic reprogramming [3, 93]. Beyond needing increased diversity of studied phage–host model systems, incorporating phages into ecosystem models will require transitioning from studying the individual phage and host towards evaluating virocells [5–7], especially when phage-specific responses impact the ecosystem (e.g., different resource acquisition, nutrient transportation or production). Such efforts will require further studying virocells molecularly and biochemically, defining functional guilds, and including both the lytic infection continuum [94] and lysogeny, since it is ubiquitous in nature [60] and can uniquely reprogram cellular metabolisms [95]. Such understanding is critical to inform phage-based applications [96, 97] and attain the predictive knowledge needed for modeling phage–host-environment interactions [10].

## Supplementary information

Supplementary information

Table S1

Table S2

Table S3

Table S4

Table S5

Supplementary Dataset

## Data Availability

The genome of *Pseudoalteromonas* sp. strain 13–15 is available at NCBI with accession numbers CP019162.1 (contig 1) and CP019163.1 (contig 2). Phage genomes are available at the Joint Genome Institute’s portal for Integrated Microbial Geomes/Virus (IMG/VR; https://img.jgi.doe.gov) under submission IDs 44760 (PSA-HP1) and 44764 (PSA-HS2), and at NCBI with ID 196895 (PSA-HP1) and 196894 (PSA-HS2). Both phage genomes’ functional annotation was improved here, and the Genbank files are found in Cyverse, together with all of the scripts used for all analyses detailed below (http://datacommons.cyverse.org/browse/iplant/home/shared/iVirus/Pseudoalteromonas_Omics). The accessions for the RNA-seq reads are in the Supplementary Dataset, Table 3. Proteomics data are available at MassIVE and the ProteomeXchange repositories with accession numbers MSV000083626 and PXD013204, respectively.
