# Supplementary material for: Phage-specific metabolic reprogramming of virocells
Source: ISME J. 2020 Jan 2;14(4):881–95. doi: 10.1038/s41396-019-0580-z (PMC7082346; doi:10.1038/s41396-019-0580-z)
Supplement: Supplementary file 1 — Supplementary information [file 41396_2019_580_MOESM1_ESM.docx]

**Phage-specific metabolic reprogramming of virocells**

**Authors:** Cristina Howard-Varona^1^, Morgan M. Lindback^2^, G. Eric Bastien^2^, Natalie Solonenko^1^, Ahmed A. Zayed^1^, HoBin Jang^1^, Bill Andreopoulos^3^, Heather M. Brewer^4^, Tijana Glavina del Rio^3^, Joshua N. Adkins^5^, Subhadeep Paul^6^, Matthew B. Sullivan*^1,7,8^ & Melissa B. Duhaime*^2^

**SUPPLEMENTARY MATERIAL**

**CONTENTS:**

1. Figure S1: RNA-Seq reads mapped to uninfected cells, phage-infected cells, and the phage genomes in every sample.
2. Figure S2: Network-based taxonomic affiliation of phages PSA-HP1 (HP1) and PSA-HS2 (HS2).
3. Figure S3: Adsorption kinetics and one-step growth curves with phages PSA-HP1 (HP1) and PSA-HS2 (HS2) independently infecting *Pseudoalteromonas* sp. strain 13-15.
4. Figure S4: RNA-seq phage genome coverage.
5. Figure S5: The complete transcriptional dynamics of phages PSA-HP1 (HP1) and PSA-HS2 (HS2).
6. Figure S6: The complete proteome dynamics of phages PSA-HP1 (HP1) and PSA-HS2 (HS2).
7. Figure S7: Phage proteins follow their corresponding transcripts.
8. Figure S8: Temporal abundance of late-expressed and structural phage genes, relative to the total proteome for each phage.
9. Figure S9: P-value bootstrap statistical test of phage-host pair codon impact.
10. Figure S10: Host proteins from the Sulfate reduction, TCA cycle and glyoxylate bypass metabolisms.
11. Figure S11: Proteome quality assessment in the uninfected host samples.
12. Figure S12: Proteome quality assessment in the HP1-virocell samples.
13. Figure S13: Proteome quality assessment in the HS2-virocell samples.
14. Table S1: Phage PSA-HP1 genes and their ‘omics classification.
15. Table S2: Phage PSA-HS2 genes and their ‘omics classification.
16. Table S3: Mean differences and significance from the ANOVA analyses on global modeling of counts from the transcriptomes and proteomes.
17. Table S4: Cosine similarity of the Relative Synonymous Codon Usage (RCSU) between phage PSA-HP1, phage PSA-HS2, and host *Pseudoalteromonas* sp. strain 13-15.
18. Table S5: Statistical significance of the codon impact between multiple phage-host pairs.
19. Supplementary Materials and Methods: Proteomics data quality assessment.
20. DataSet (Spreadsheet attached).
21. Supplementary references.


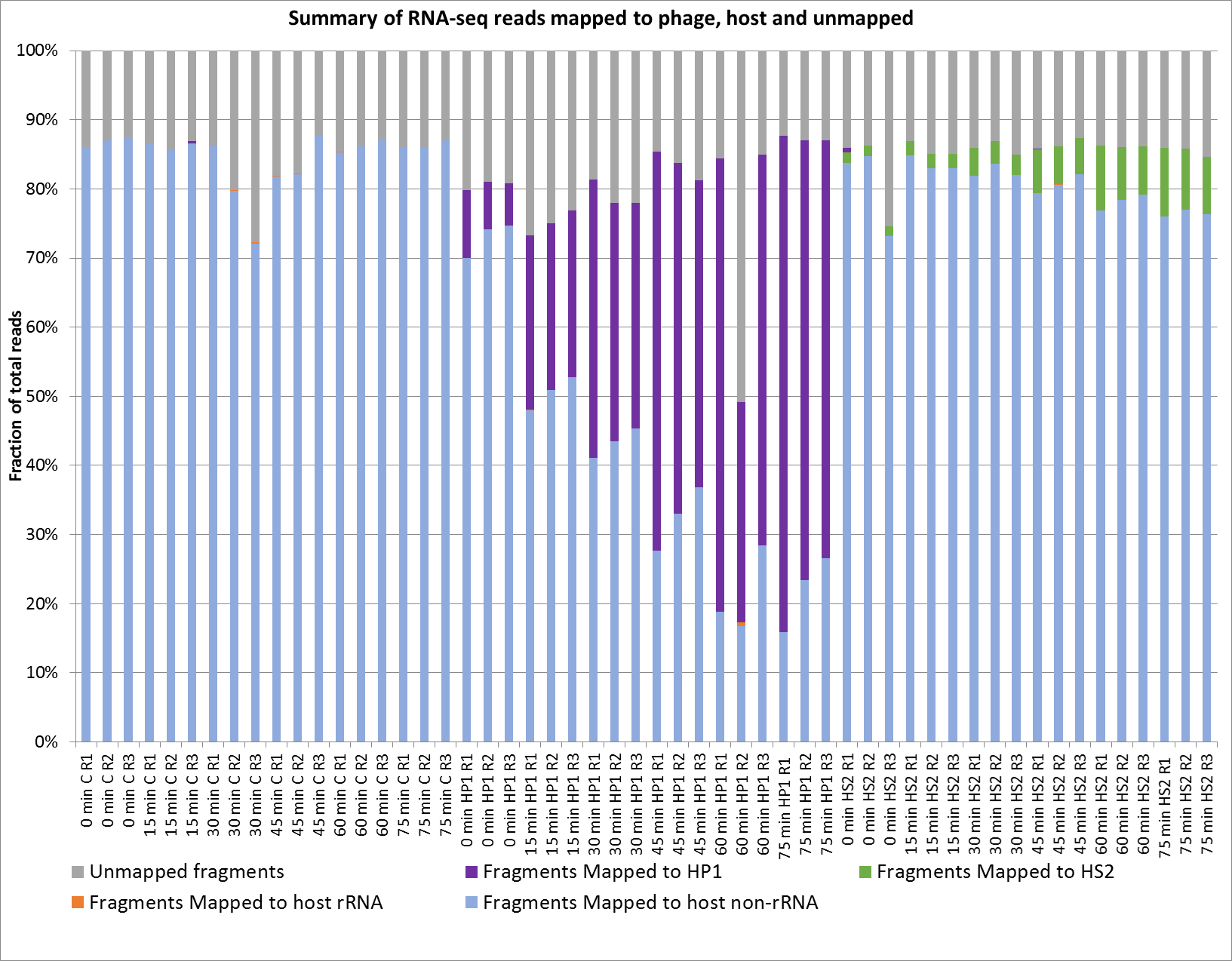


**Figure S1: RNA-Seq reads mapped to uninfected cells, phage-infected cells, and the phage genomes in every sample**. Represented is the fraction of reads mapped to the host (either to the rRNA or to the remaining of the genome), to phage HP1, to phage HS2, or unmapped, in either of the treatments: uninfected control samples (“C’), HP1-infected cells (“HP1”) or HS2-infected cells (“HS2”). The time point (in min) begins at 0 minutes, which indicates 15 minutes post phage-addition. The ‘R’ indicates the biological replicate. Host genome coverage (blue) ranges from 86x to 757x. Phage HP1 genome coverage (purple) ranges from ~4800x to ~38000x. Phage HS2 genome coverage (green) ranges from ~717x to ~8700x.


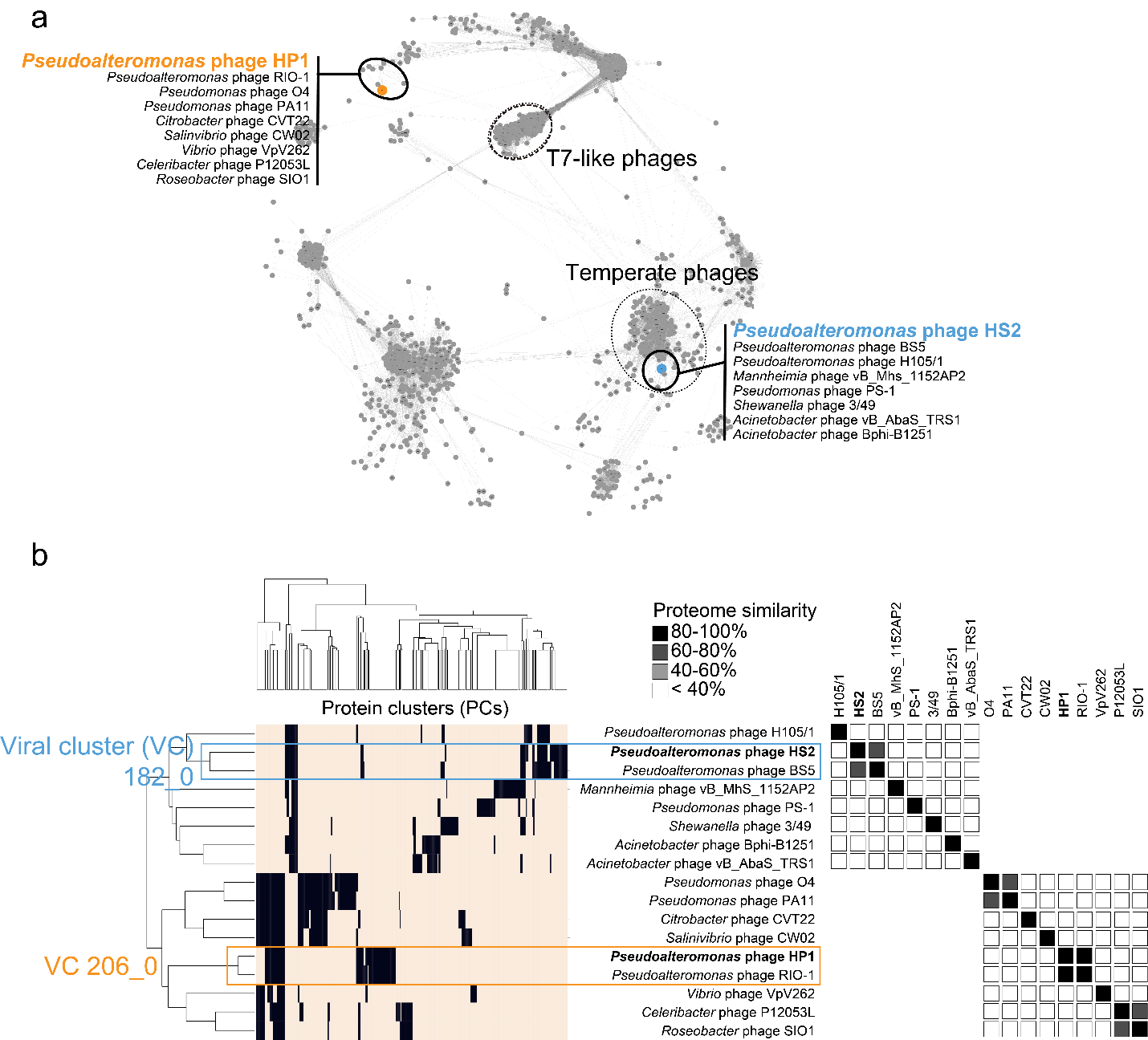


**Figure S2: Network-based taxonomic affiliation of phages PSA-HP1 (HP1) and PSA-HS2 (HS2).** (A) Nodes indicate phage genomes and edges between two nodes indicate their statistically weighted pairwise similarities with genome-genome similarity score of ≥1. There are 1,443 nodes and 52,145 edges in this network. For clarity, the genomes that do not link to HP1 and HS2 were excluded. Eight and Seven phages that link to HP1 and HS2 are represented, respectively. (b) Left, module profile showing the presence and absence of shared protein clusters (PCs, gray blocks) between virus genomes, which directly link to HP1 or HS2 on the network. Each row depicts a phage and each column represents a PC, respectively. Viral clusters (VCs) that contain phages HP1 and HS2 and their closest relatives are highlighted, respectively. Right, the columns and rows correspond to the phages. Each cell indicates the percentage of shared PCs (proteome similarity) between two phages. Color tags are used to visualize the genomic relatedness based on the fraction of shared PCs. For details, see DataSet.

**
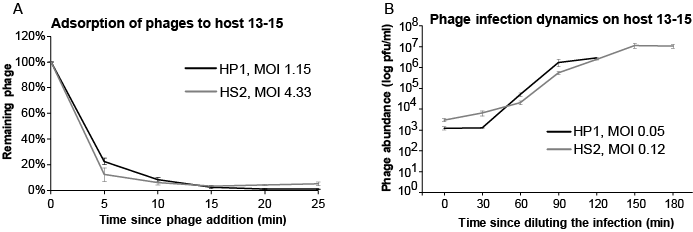
**

**Figure S3: Adsorption kinetics and one-step growth curves with phages PSA-HP1 (HP1) and PSA-HS2 (HS2) independently infecting *Pseudoalteromonas* sp. strain 13-15.** (A) Adsorption at MOI of 1 for phage HP1 and 4 for phage HS2. (B) One-step growth curves at MOI of 0.05 for phage HP1 and 0.1 for phage HS2. Time 0 minutes represents the first sample after a 1:100 dilution done after 15 minutes of phage-host adsorption. For both graphs, the average of three biological replicates and their error is represented.


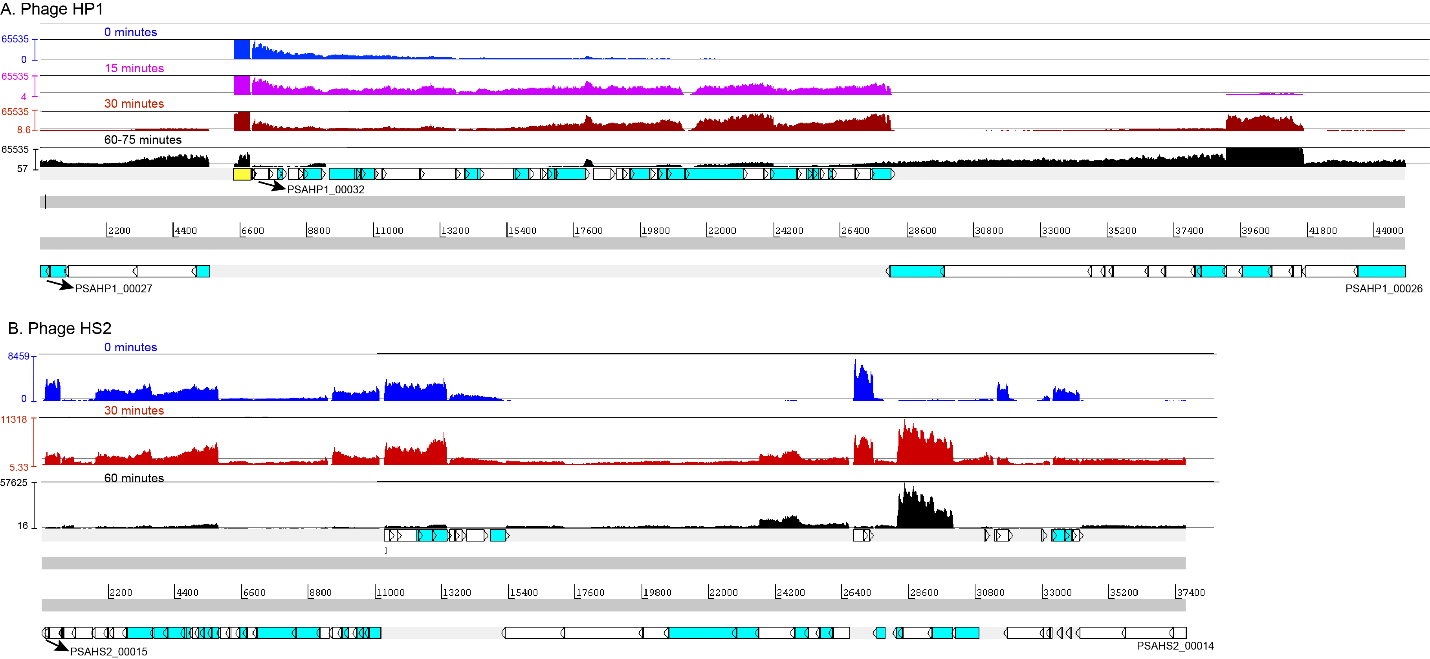


**Figure S4: RNA-seq phage genome coverage.** Reads mapped to each of the phage genomes (open reading frames in neon blue and white as generated via Artemis(1)) and temporally separated by colors. The yellow indicates a non-coding region with the highest number of reads mapped to it, representing a putative non-coding RNA.


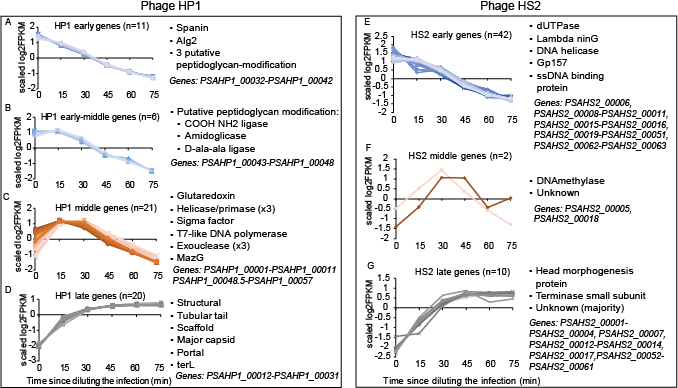


**Figure S5: The complete transcriptional dynamics of phages PSA-HP1 (HP1) and PSA-HS2 (HS2)**. Represented is the scaled log2FPKM transcript abundances for all genes in each temporal category throughout the course of the infection. The ‘n’ represents the number of genes plotted. Gene ID and salient functions for each category are on the right of each graph. Time 0 min indicates 15 minutes post diluting the infection. Similar to previously characterized cyanophages(2), the *Pseudoalteromonas* phages express their genes in early, middle and late categories that can be further temporally resolved.


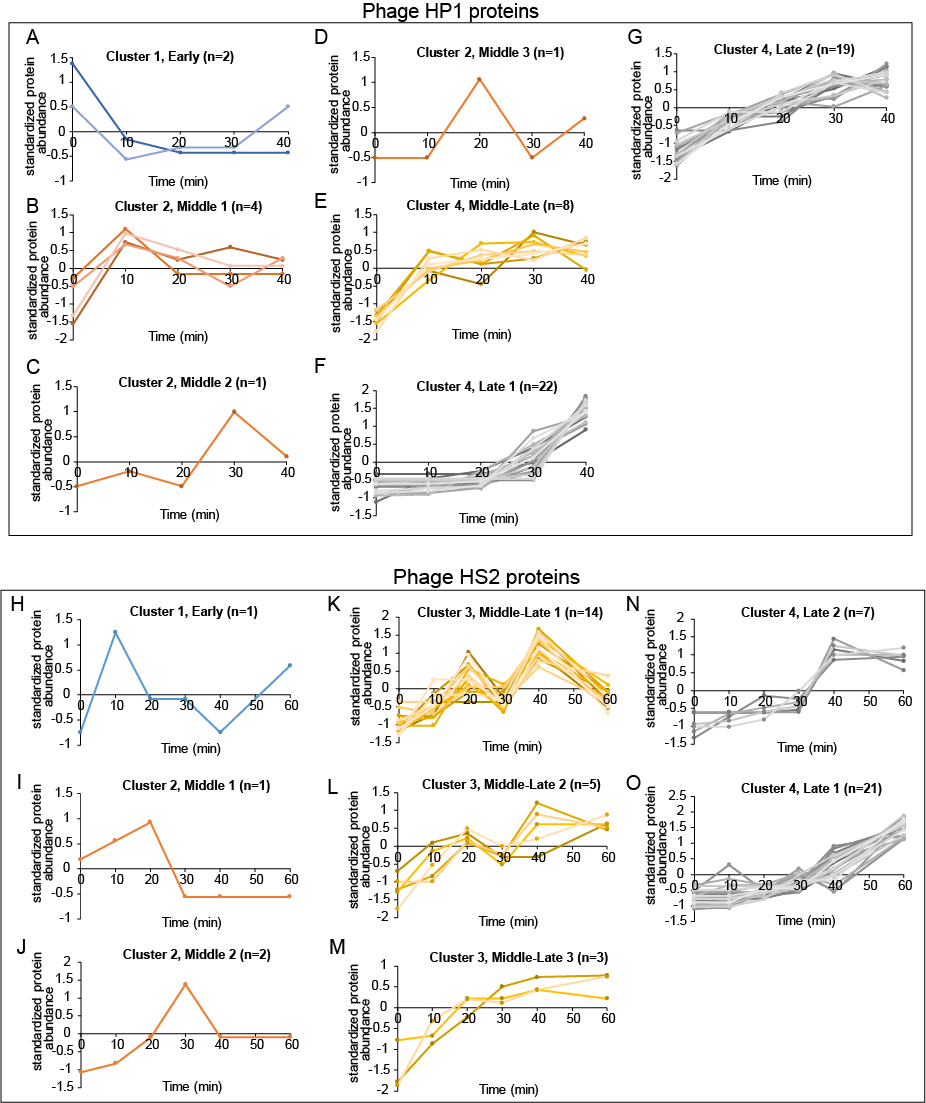


**Figure S6: The complete proteome dynamics of phages PSA-HP1 (HP1) and PSA-HS2 (HS2).** Represented in the graphs is the z-score (standardized) protein abundances for all the proteins in each temporal group throughout the infection. Time 0 minutes indicates 15 minutes after diluting the infection.

**
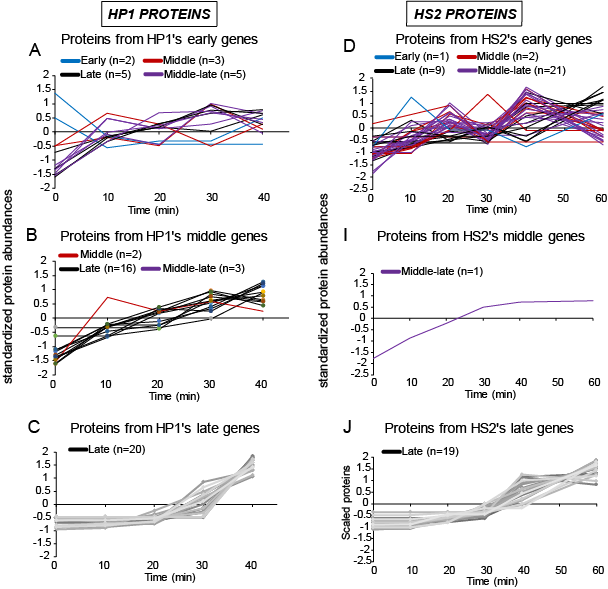
**

**Figure S7: Phage proteins follow their corresponding transcripts.** Represented in the graphs is the z-score (standardized) protein abundances for all the proteins detected for the early, middle or late transcripts. Time 0 minutes indicates 15 minutes after diluting the infection.


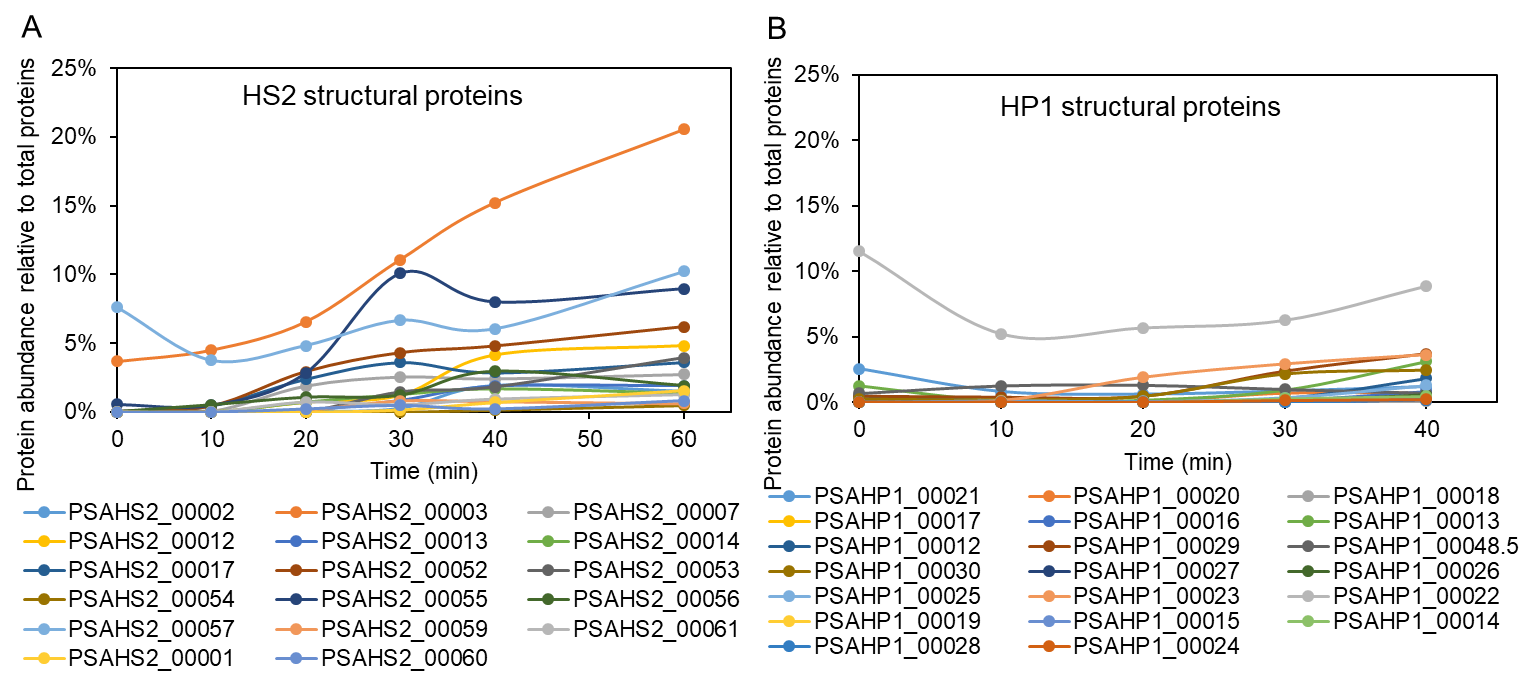


**Figure S8: Temporal abundance of late-expressed and structural phage genes, relative to the total proteome for each phage.** The late-expressed genes, except those annotated as involved in lysis, and other non-late-expressed structural proteins are included. The relative abundance was calculated for each individual protein (relative to total protein abundances) of phage HS2 (A) or HP1 (B).

**
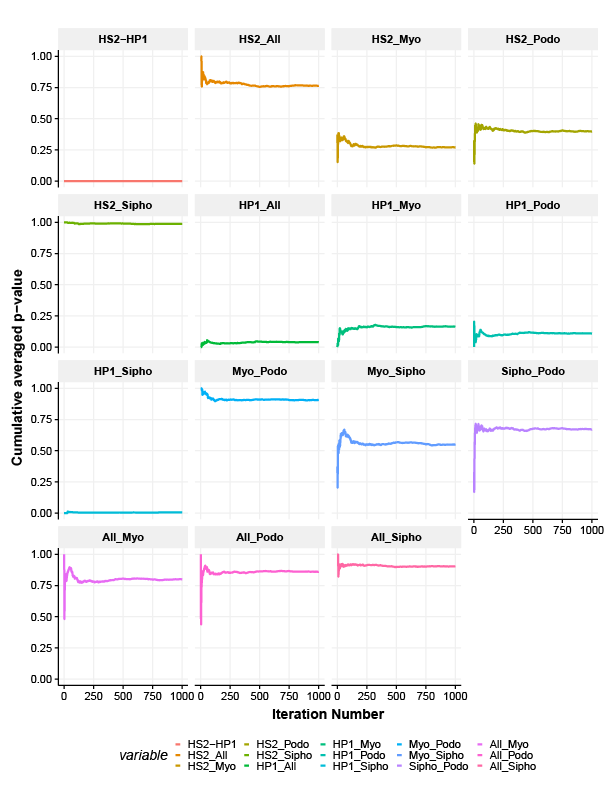
**

**Figure S9: P-value bootstrap statistical test of phage-host pair codon impact.** Boostrapping of p-value over 1000 iterations to determine if there is significant difference in phage-host pair codon impact between HS2, HP1, Myoviridae, Podoviridae and Siphoviridae groups. There is significant difference (pairwise t-test with adjusted p-value Holm-Bonferri) between HS2-HP1 (P-value = 0.0002), HP1-All (P-value = 0.0330), and HP1-Sipho (P-value = 0.0075).


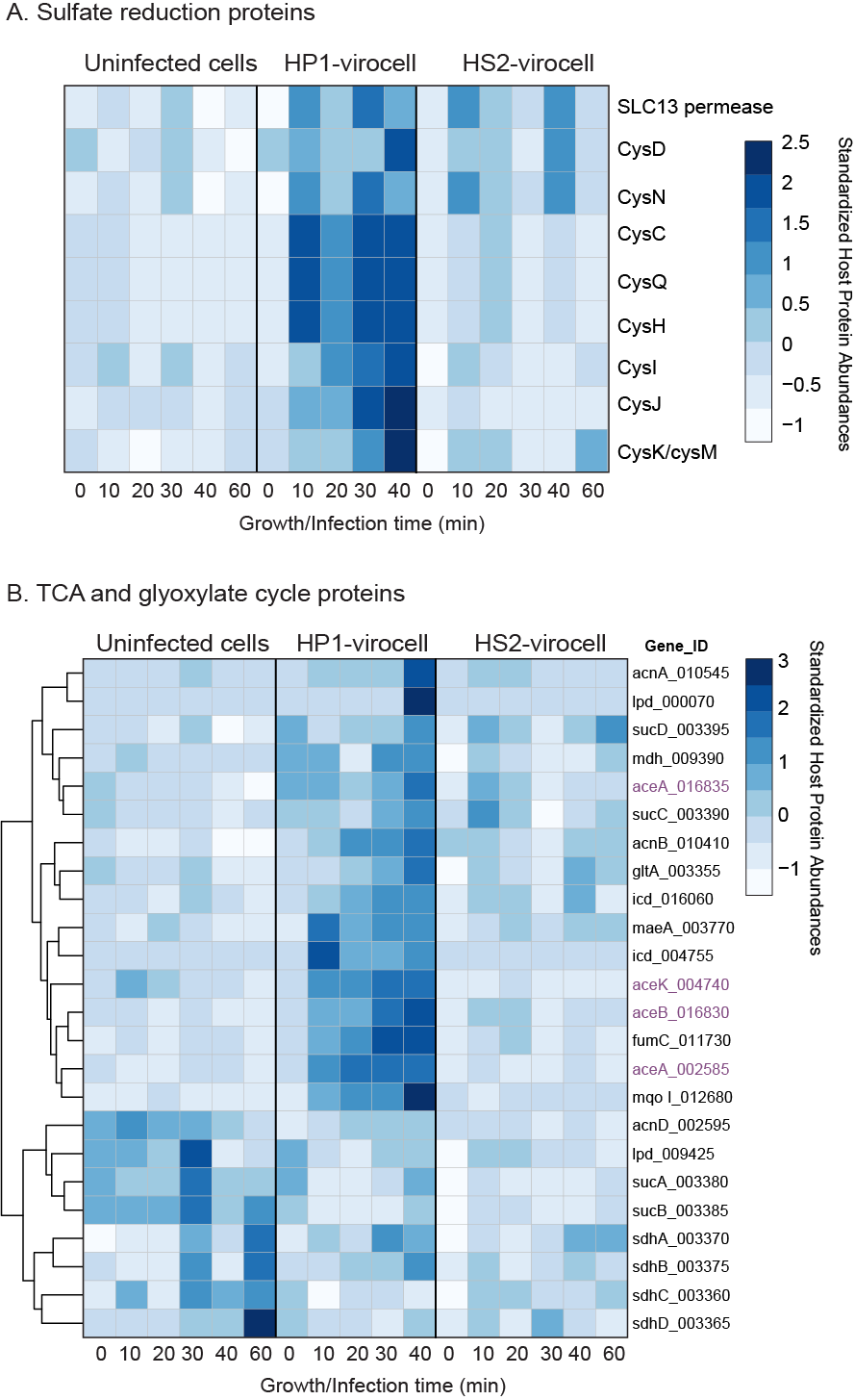


**Figure S10: Host proteins from the Sulfate reduction, TCA cycle and glyoxylate bypass metabolisms. (A)** Sulfate transport and reduction proteins. **(B)** TCA cycle and glyoxylate bypass proteins. For both, the heatmap represents the z-score (standardized) protein values for the genes represented in Figure 5, clustered according to their abundance patterns (in B only).


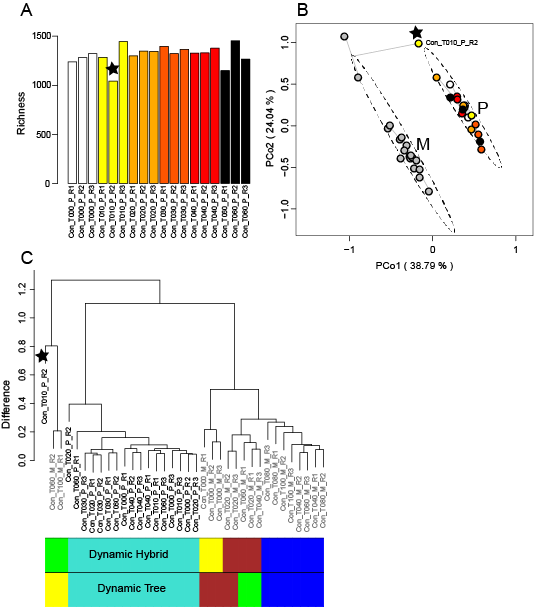


**Figure S11: Proteome quality assessment in the uninfected host samples.** (A) Representation of the richness (number of distinct proteins) per sample collected shows which samples have fewer total proteins than the other samples in the same time point group. A clear example of lower richness is the uninfected control sample taken at 10 minutes post diluting the infection (T010) from biological replicate 2 (R2). (B) Principal Coordinate Analysis on a Bray-Curtis dissimilarity matrix of the proteomes shows that few samples fall outside the 95% confidence intervals (dashed ellipses) drawn around the centroid of each group. The “P” group is the group of “proteomes” used in this study colored by their sampling time as indicated in panel (A). The “M” group is a different proteome dataset used as decoy for testing the within-group similarity of this study’s proteomes. The Con_T010_P_R2 sample is the most distinct as it falls outside the 95% confidence intervals. (C) Hierarchical clustering on a Bray-Curtis dissimilarity matrix of the proteomes using both the Dynamic tree cutting and the Dynamic Hybrid tree cutting methods shows that the Con_T010_P_R2 sample clustered outside of the “P” group of proteomes. The colors represent the different clustering groups assigned by each method.

**
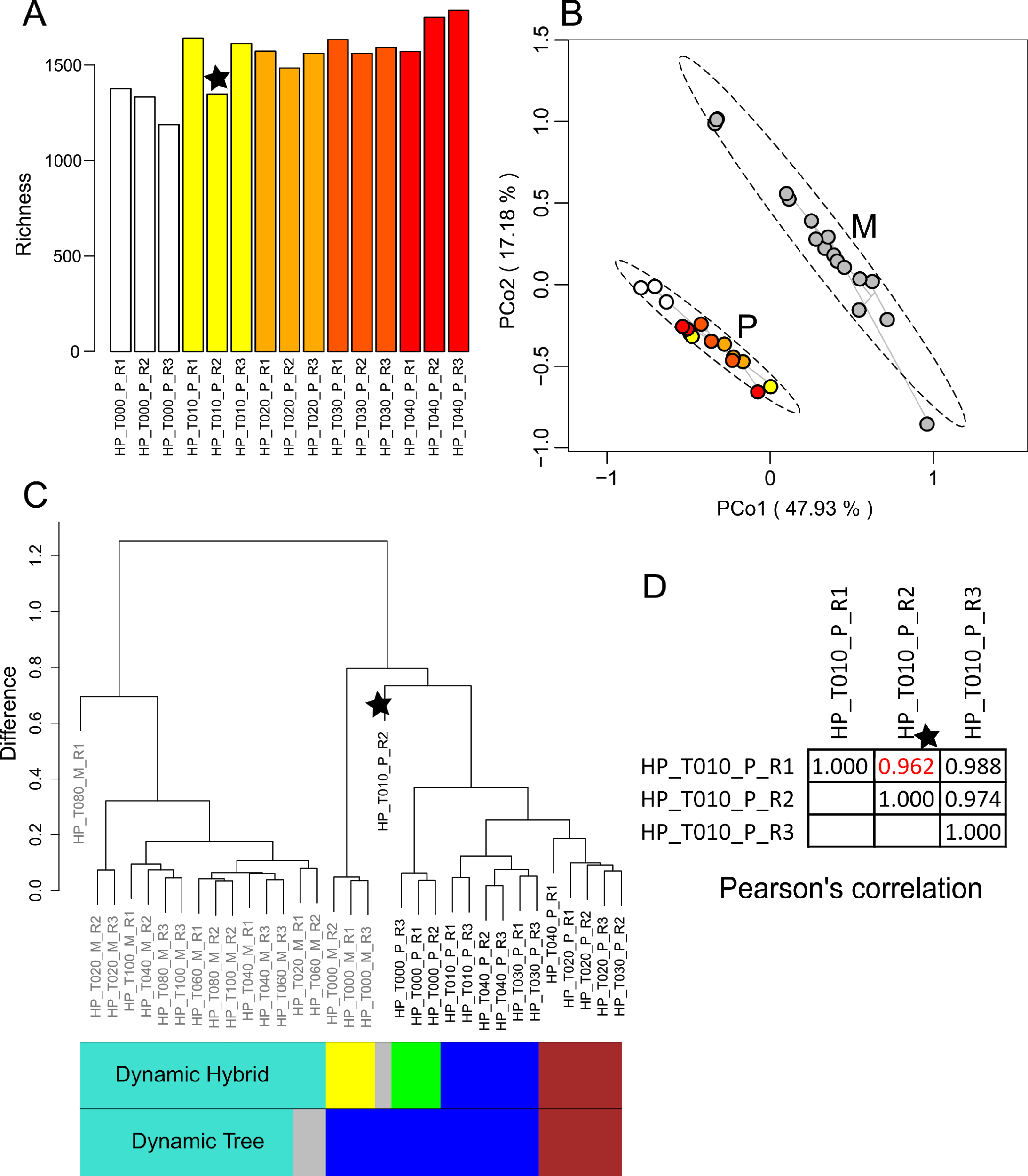
**

**Figure S12: Proteome quality assessment in the HP1-virocell samples.** (A) Representation of the richness (number of distinct proteins) per sample collected shows which samples have fewer total proteins than the other samples in the same time point group. A clear example of lower richness is the HP1-infected sample (HP) taken at 10 minutes post diluting the infection (T010) from biological replicate 2 (R2). (B) Principal Coordinate Analysis on a Bray-Curtis dissimilarity matrix of the proteomes with the 95% confidence intervals (dashed ellipses) drawn around the centroid of each group. The “P” group is the group of “proteomes” used in this study colored by their sampling time as indicated in panel (A). The “M” group is a different proteome dataset used as decoy for testing the within-group similarity of this study’s proteomes. (C) Hierarchical clustering on a Bray-Curtis dissimilarity matrix of the proteomes using both the Dynamic tree cutting method and the Dynamic Hybrid tree cutting methods. The colors represent the different clustering groups assigned by each method. The Dynamic Hybrid tree cutting method shows that the HP_T010_P_R2 sample does not cluster with the rest of the samples in its “P” group. (D) Pairwise pearson’s correlations between the replicates of the only HP1-virocell proteome sample that was excluded from downstream analyses (“HP_T010_P_R2”, highlighted by a star in panels (A, C, and D)). Red text indicates a correlation that is less than 0.97.

**
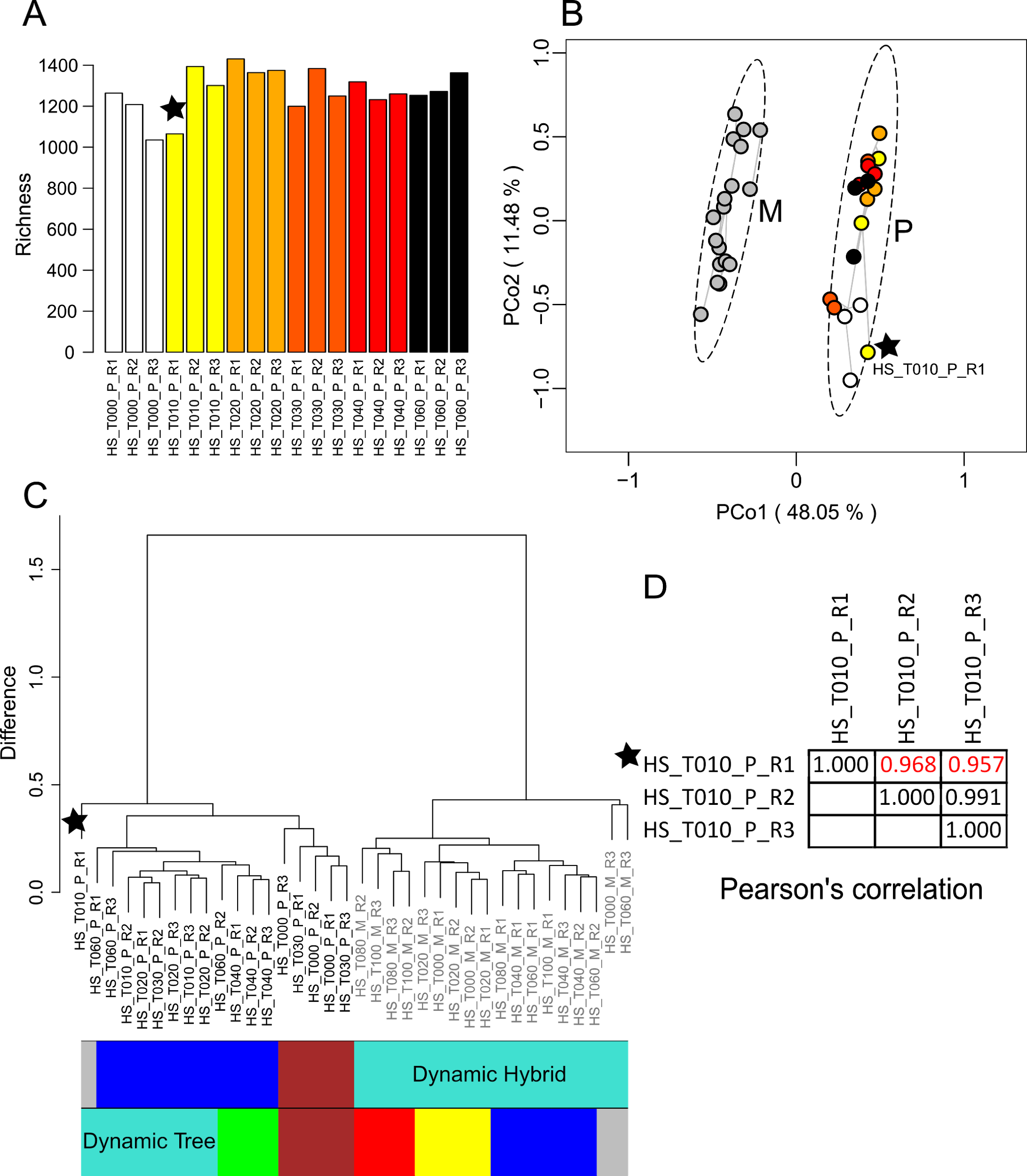
**

**Figure S13: Proteome quality assessment in the HS2-virocell samples.** (A) Representation of the richness (number of distinct proteins) per sample collected shows which samples have fewer total proteins than the other samples in the same time point group. A clear example of lower richness is the HS2-infected sample (“HS”) taken at 10 minutes post diluting the infection (T010) from biological replicate 1 (R1). (B) Principal Coordinate Analysis on a Bray-Curtis dissimilarity matrix of the proteomes shows that few samples fall outside the 95% confidence intervals (dashed ellipses) drawn around the centroid of each group. The “P” group is the group of “proteomes” used in this study colored by their sampling time as indicated in panel (A). The “M” group is a different proteome dataset used as decoy for testing the within-group similarity of this study’s proteomes. The HS_T010_P_R1 sample is the most distinct as it falls outside the 95% confidence intervals. (C) Hierarchical clustering on a Bray-Curtis dissimilarity matrix of the proteomes using both the Dynamic tree cutting and the Dynamic Hybrid tree cutting methods. The colors represent the different clustering groups assigned by each method. The Dynamic Hybrid tree cutting method shows that the HS_T010_P_R1 sample does not cluster with the rest of its group of samples. (D) Pairwise pearson’s correlations between the replicates of the only uninfected-host proteome sample (HS_T010_P_R1) that was excluded from downstream analyses (highlighted by a star in panels (A-D)). Red text indicates a correlation that is less than 0.97.

**Table S1: Phage PSA-HP1 genes and their ‘omics classification**. Phage HP1 gene ID (first column), the strand from which its transcribed (second column), its coordinates (start and stop, third and fourth columns, respectively), its annotation (fifth column), and the temporal cluster (or subcluster, for the transcripts) that the transcript (sixth and seventh columns) or the protein (eight column) belongs to. The temporal clusters are mainly assigned based on k-means and then they are manually inspected by plotting their z-score transformed log2FPKM values (for the transcripts) or the z-score transformed raw counts (for the proteins). Such values are in tab 14 of the supplementary Dataset.

| **ID** | **Strand** | **start (bp)** | **stop (bp)** | **Final annotation** | **Transcription temporal cluster** | **Transcription temporal subcluster** | **Protein temporal cluster** |
| --- | --- | --- | --- | --- | --- | --- | --- |
| PSAHP1_00001 | sense | 21271 | 23187 | T7-like DNAP (family A) | Middle | Middle | Late 2 |
| PSAHP1_00002 | sense | 23200 | 23856 | putative ssDNA-binding protein | Middle | Middle | Late 2 |
| PSAHP1_00003 | sense | 23856 | 24083 | unknown function | Middle | Middle | Late 1 |
| PSAHP1_00004 | sense | 24092 | 24961 | 5'-3' exonuclease | Middle | Middle | Late 2 |
| PSAHP1_00005 | sense | 24954 | 25301 | MazG ppGpp pyrophosphohydrolase | Middle | Middle | Late 2 |
| PSAHP1_00006 | sense | 25288 | 25479 | unknown function | Middle | Middle | Late 2 |
| PSAHP1_00007 | sense | 25476 | 25706 | unknown function | Middle | Middle | Late 1 |
| PSAHP1_00008 | sense | 25696 | 26136 | Endonuclease VII | Middle | Middle | Late 2 |
| PSAHP1_00008.5 | sense | 25700 | 25978 | unknown function | - | - | Middle 1 |
| PSAHP1_00009 | sense | 26133 | 26888 | Proofreading exonuclease; DNAP III alpha | Middle | Middle | Late 2 |
| PSAHP1_00010 | sense | 26888 | 27370 | Lysozyme | Middle | Middle | Late 2 |
| PSAHP1_00011 | sense | 27453 | 28052 | unknown function | Middle | Middle | Late 2 |
| PSAHP1_00012 | antisense | 28053 | 29834 | Structural | Late | Late | Late 1 |
| PSAHP1_00013 | antisense | 29834 | 34657 | Structural | Late | Late | Late 1 |
| PSAHP1_00014 | antisense | 34670 | 35101 | unknown function | Late | Late | Late 1 |
| PSAHP1_00015 | antisense | 35103 | 35393 | unknown function | Late | Late | Late 1 |
| PSAHP1_00016 | antisense | 35390 | 36490 | T7-like Tubular tail B | Late | Late | Late 1 |
| PSAHP1_00017 | antisense | 36573 | 37115 | Structural | Late | Late | Late 1 |
| PSAHP1_00018 | antisense | 37124 | 38086 | T7-like Tubular tail B | Late | Late | Late 1 |
| PSAHP1_00019 | antisense | 38086 | 38304 | unknown function | Late | Late | Late 1 |
| PSAHP1_00020 | antisense | 38304 | 39056 | Tail tubular protein A | Late | Late | Late 1 |
| PSAHP1_00021 | antisense | 39140 | 39658 | Structural | Late | Late | Late 1 |
| PSAHP1_00022 | antisense | 39667 | 40623 | Major capsid | Late | Late | Late 1 |
| PSAHP1_00023 | antisense | 40634 | 41320 | Scaffold | Late | Late | Late 1 |
| PSAHP1_00024 | antisense | 41313 | 41594 | unknown function | Late | Late | Late 1 |
| PSAHP1_00025 | antisense | 41757 | 43457 | Portal | Late | Late | Late 1 |
| PSAHP1_00026 | antisense | 43454 | 45034 | Terminase large subunit | Late | Late | Late 1 |
| PSAHP1_00027 | antisense | 8 | 301 | Structural | Late | Late | Late 1 |
| PSAHP1_00028 | antisense | 303 | 824 | unknown function | Late | Late | Late 1 |
| PSAHP1_00029 | antisense | 936 | 3179 | Tailspike | Late | Late | Late 1 |
| PSAHP1_00030 | antisense | 3208 | 5148 | Structural | Late | Late | Late 1 |
| PSAHP1_00031 | antisense | 5157 | 5567 | Putative holin of 3 TMs | Late | Late | Late 1 |
| PSAHP1_00032 | sense | 7048 | 7530 | unknown function | Early | Early | Middle 1 |
| PSAHP1_00033 | sense | 7584 | 7829 | hypothetical protein | Early | Early | Late 2 |
| PSAHP1_00034 | sense | 7839 | 7991 | ribosome; protein-protein interaction | Early | Early | Early |
| PSAHP1_00035 | sense | 8190 | 8525 | unknown function | Early | Early | Middle 2 |
| PSAHP1_00036 | sense | 8510 | 8671 | unknown function | Early | Early | #N/A |
| PSAHP1_00037 | sense | 8668 | 9237 | Spanin | Early | Early | Early |
| PSAHP1_00038 | sense | 9557 | 10402 | unknown function | Early | Early | Late 2 |
| PSAHP1_00039 | sense | 10393 | 10572 | hypothetical protein | Early | Early | #N/A |
| PSAHP1_00040 | sense | 10581 | 10991 | gamma-flu cyclotransferase (AlG2) | Early | Early | Middle-Late |
| PSAHP1_00041 | sense | 11091 | 11267 | putative DNA packaging | Early | Early | Middle 1 |
| PSAHP1_00042 | sense | 11267 | 12520 | glutamine amidotransferase | Early | Early | Middle-late |
| PSAHP1_00043 | sense | 12517 | 13716 | COOH NH2 ligase | Early | Early-Middle | Late 2 |
| PSAHP1_00044 | sense | 13713 | 14000 | unknown function | Early | Early-Middle | Middle-Late |
| PSAHP1_00045 | sense | 13993 | 14487 | unknown function | Early | Early-Middle | Late 2 |
| PSAHP1_00046 | sense | 14444 | 15673 | Amidoglicase | Early | Early-Middle | Late 2 |
| PSAHP1_00047 | sense | 15618 | 16121 | ATP-grasp (D-ala D-ala ligase) | Early | Early-Middle | Middle-late |
| PSAHP1_00048 | sense | 16114 | 16533 | Transcription elongation factor SPT5 | Early | Early-Middle | Middle-Late |
| PSAHP1_00048.5 | sense | 16521 | 16718 | Putative portal protein | Middle | Middle | Middle-Late |
| PSAHP1_00049 | sense | 16718 | 16939 | glutaredoxin | Middle | Middle | Late 2 |
| PSAHP1_00050 | sense | 17013 | 17963 | helicase/primase gp50 | Middle | Middle | Middle-Late |
| PSAHP1_00051 | sense | 18269 | 18802 | helicase/primase gp51 | Middle | Middle | Late 2 |
| PSAHP1_00052 | sense | 19033 | 19212 | helicase/primase gp52 | Middle | Middle | Middle 1 |
| PSAHP1_00053 | sense | 19205 | 19462 | Putative exonuclease | Middle | Middle | Late 2 |
| PSAHP1_00054 | sense | 19459 | 20121 | Putative sigma factor s70 | Middle | Middle | Late 2 |
| PSAHP1_00055 | sense | 20114 | 20368 | unknown function | Middle | Middle | Late 2 |
| PSAHP1_00056 | sense | 20368 | 20664 | unknown function | Middle | Middle | Middle-late |
| PSAHP1_00057 | sense | 20664 | 21269 | Putative T7-like DNAP | Middle | Middle | Middle 3 |

**Table S2: Phage PSA-HS2 genes and their ‘omics classification.** Phage HS2 gene ID (first column), its coordinates (start and stop, second and third columns, respectively), its gene length (fourth column), the strand from which it's transcribed (fifth column), its annotation (sixth column), and the temporal cluster that the transcript (seventh and eight columns) or the protein (ninth column) belongs to. The temporal clusters are mainly assigned based on k-means and then they are manually inspected by plotting their z-score transformed log2FPKM values (for the transcripts) or the z-score transformed raw counts (for the proteins). Such values are in tab 14 of the supplementary Dataset.

| **Gene_ID** | **start** | **end** | **geneLength** | **strand** | Final annotation | **Transcription temporal cluster** | **Protein temporal cluster** |
| --- | --- | --- | --- | --- | --- | --- | --- |
| PSAHS2_00001 | 27545 | 27790 | 246 | - | unknown function | Late | Late 1 |
| PSAHS2_00002 | 28208 | 28387 | 180 | - | Putative tail component | Late | Late 2 |
| PSAHS2_00003 | 28400 | 29353 | 954 | - | Putative major capsid protein | Late | Late 1 |
| PSAHS2_00004 | 29366 | 30022 | 657 | - | Cell wall hydrolase | Late | Late 2 |
| PSAHS2_00005 | 30153 | 30911 | 759 | - | DNA modification methylase | Middle | Middle-late 3 |
| PSAHS2_00006 | 31494 | 31883 | 390 | + | YopX protein | Early | 0 |
| PSAHS2_00007 | 31880 | 33007 | 1128 | - | putative head morphogenesis protein, SPP1 | Late | Late 1 |
| PSAHS2_00008 | 33062 | 33235 | 174 | - | putative glycine-rich cell wall structural protein 1 precursor | Early | Middle-late 1 |
| PSAHS2_00009 | 33354 | 33725 | 372 | + | unknown function | Early | 0 |
| PSAHS2_00010 | 33747 | 33992 | 246 | + | unknown function | Early | 0 |
| PSAHS2_00011 | 33979 | 34194 | 216 | + | Putative tail host specificity or injection protein | Early | Middle-late 1 |
| PSAHS2_00012 | 34252 | 35745 | 1494 | - | Portal protein | Late | Late 1 |
| PSAHS2_00013 | 35749 | 37320 | 1572 | - | Putative terminase large subunit | Late | Late 1 |
| PSAHS2_00014 | 37317 | 37727 | 411 | - | Terminase small subunit | Late | Late 1 |
| PSAHS2_00015 | 110 | 241 | 132 | - | Putative aminofutalosine synthase MqnE | Early | 0 |
| PSAHS2_00016 | 238 | 636 | 399 | - | unknown function | Early | Late 1 |
| PSAHS2_00017 | 710 | 1099 | 390 | - | Putative structural | Late | Late 1 |
| PSAHS2_00018 | 1123 | 1668 | 546 | - | unknown function | Middle | 0 |
| PSAHS2_00019 | 1782 | 2165 | 384 | - | deoxyuridine 5'-triphosphate nucleotidohydrolase (dUTPase) | Early | Middle-late 2 |
| PSAHS2_00020 | 2175 | 2366 | 192 | - | Arabinose operon regulatory protein | Early | 0 |
| PSAHS2_00021 | 2360 | 2791 | 432 | - | Putative P22 integrase | Early | Middle-late 2 |
| PSAHS2_00022 | 2794 | 3645 | 852 | - | Putative phosphoadenosine phosphosulfate reductase (CysH) | Early | Middle-late 1 |
| PSAHS2_00023 | 3715 | 4161 | 447 | - | ninB | Early | Middle-late 1 |
| PSAHS2_00024 | 4148 | 4735 | 588 | - | Bacteriophage Lambda NinG recombination protein | Early | Late 2 |
| PSAHS2_00025 | 4722 | 4883 | 162 | - | unknown function | Early | 0 |
| PSAHS2_00026 | 4990 | 5133 | 144 | - | Putative glycoside hydrolase (lysozyme) | Early | 0 |
| PSAHS2_00027 | 5170 | 5346 | 177 | - | Repressor C2 | Early | 0 |
| PSAHS2_00028 | 5347 | 5592 | 246 | - | unknown function | Early | Middle-late 2 |
| PSAHS2_00029 | 5597 | 5827 | 231 | - | Putative AAA family ATPase | Early | Middle-late 1 |
| PSAHS2_00030 | 5910 | 6212 | 303 | - | unknown function | Early | Late 1 |
| PSAHS2_00031 | 6205 | 6534 | 330 | - | Putative NinX | Early | Late 2 |
| PSAHS2_00032 | 6534 | 6752 | 219 | - | Putative LuxR transcriptional regulator | Early | Late 1 |
| PSAHS2_00033 | 6752 | 7075 | 324 | - | Protein of unknown function | Early | Late 1 |
| PSAHS2_00034 | 7075 | 8382 | 1308 | - | DnaB-like helicase | Early | Middle-late 1 |
| PSAHS2_00035 | 8379 | 9149 | 771 | - | DNA replication protein | Early | Middle-late 3 |
| PSAHS2_00036 | 9201 | 9452 | 252 | - | putative glycine-rich cell wall structural protein 1 precursor | Early | Late 2 |
| PSAHS2_00037 | 9587 | 9883 | 297 | - | putative signal peptide | Early | Middle-late 1 |
| PSAHS2_00038 | 9895 | 10110 | 216 | - | putative ABC-F family ATP-binding cassette | Early | Middle-late 1 |
| PSAHS2_00039 | 10107 | 10394 | 288 | - | unknown function | Early | Middle-late 1 |
| PSAHS2_00040 | 10394 | 10588 | 195 | - | Putative lambda integration | Early | Middle-late 1 |
| PSAHS2_00041 | 10588 | 10794 | 207 | - | Putative P22 Eaa lysogeny protein | Early | 0 |
| PSAHS2_00042 | 10785 | 11153 | 369 | - | Putative lambda exclusion protein | Early | Middle-late 1 |
| PSAHS2_00043 | 11311 | 11472 | 162 | + | Putative lambda recombination protein bet | Early | Middle-late 2 |
| PSAHS2_00044 | 11484 | 11708 | 225 | + | Putative lambda endolysin | Early | Middle 2 |
| PSAHS2_00045 | 11719 | 12399 | 681 | + | DNA repair protein | Early | Middle-late 1 |
| PSAHS2_00046 | 12392 | 12874 | 483 | + | Siphovirus Gp157 replication module | Early | Middle-late 3 |
| PSAHS2_00047 | 12884 | 13357 | 474 | + | Single-stranded DNA-binding protein | Early | Middle-late 2 |
| PSAHS2_00048 | 13465 | 13623 | 159 | + | Putative lambda holin | Early | Middle 1 |
| PSAHS2_00049 | 13620 | 13835 | 216 | + | Putative tail host specificity protein | Early | Delayed early |
| PSAHS2_00050 | 14037 | 14555 | 519 | + | Lysozyme | Early | Late 1 |
| PSAHS2_00051 | 14797 | 15270 | 474 | + | Holin of 3TMs (DUF3154) | Early | Late 1 |
| PSAHS2_00052 | 15301 | 17232 | 1932 | - | Tailspike | Late | Late 1 |
| PSAHS2_00053 | 17232 | 19835 | 2604 | - | Receptor recognition | Late | Late 1 |
| PSAHS2_00054 | 19835 | 20668 | 834 | - | Muskelin; jelly-roll | Late | Late 1 |
| PSAHS2_00055 | 20668 | 22905 | 2238 | - | Tail tape measure protein | Late | Late 1 |
| PSAHS2_00056 | 22902 | 23618 | 717 | - | Putative pre-tape measure chaperone protein | Late | Late 2 |
| PSAHS2_00057 | 23671 | 24828 | 1158 | - | Tail tube protein | Late | Late 1 |
| PSAHS2_00058 | 24831 | 25289 | 459 | - | Putative hydrolase | Late | Late 1 |
| PSAHS2_00059 | 25286 | 25675 | 390 | - | Putative HK97 gp10 virion protein | Late | Late 2 |
| PSAHS2_00060 | 25675 | 26091 | 417 | - | unknown function | Late | Late 1 |
| PSAHS2_00061 | 26091 | 26633 | 543 | - | Portal protein | Late | Late 1 |
| PSAHS2_00062 | 26771 | 27094 | 324 | + | unknown function | Early | Middle-late 1 |
| PSAHS2_00063 | 27094 | 27279 | 186 | + | Putative restriction alleviation protein | Early | Middle-late 1 |

**Table S3: Mean differences and statistical significance from the ANOVA analyses on the global modeling of counts from the transcriptomes and proteomes.** Statistical parameters from the ANOVA analyses performed to create Figure 2. The ANOVA was run on the transcript ('Transcriptomics') or protein ('Proteomics') raw counts. The model is explained in the methods, but briefly, it tries to find what variables best explain the trend in the counts, either the treatment ('host type', which indicates if it's uninfected or infected with either phage), the temporal dynamics ('time') or the interaction between both.


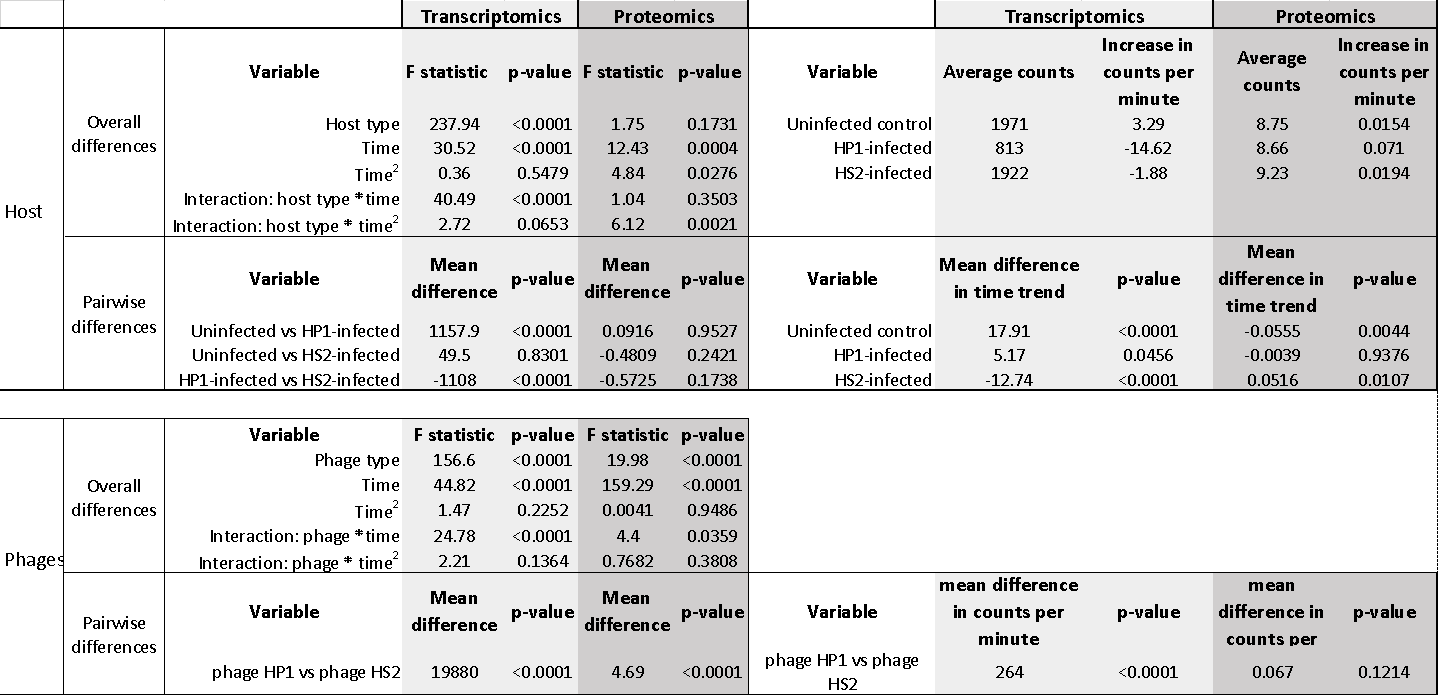


**Table S4: Cosine similarity of the Relative Synonymous Codon Usage (RSCU) between phage PSA-HP1, phage PSA-HS2, and host *Pseudoalteromonas* sp. strain 13-15.** The cosine similarity measures the cosine of the angle between two non-zero vectors, to determine how similar those two vectors are. Here, the vectors are the RCSU values of the host and the two phages. The RSCU values are the number of times a particular codon is observed, relative to the number of times that the codon would be observed for a uniform synonymous codon usage (i.e. all the codons for a given amino-acid have the same probability) in the absence of any codon usage bias. A codon that is used less frequently than expected will have an RSCU value of less than 1.00, and vice versa for a codon that is used more frequently than expected. The RSCU vectors are of length 64, because they are 64 possible codons. The cosine similarity between the RSCU vectors is a metric that determines how similar the codon bias is between each pair of genomes, and is represented in the first column of the table. In the second column is the cosine distance, which is 1 minus the cosine similarity.

|  | Cosine similarity of RSCU vector | Cosine distance RSCU vectors |
| --- | --- | --- |
| HP1-host | 0.9116732 | 0.088 |
| HS2-host | 0.9887168 | 0.011 |
| HP1-HS2 | 0.8622574 | 0.138 |

**Table S5: Statistical significance of the codon impact between multiple phage-host pairs.** From RefSeq, 1185 phage-host pairs with complete genomes were downloaded on June 2019 (229 pairs for Myoviridae, 166 pairs for Podoviridae, and 671 pairs for Siphoviridae). For each family, a codon impact vector was created with 64 (total possible codons) times the number of pairs (e.g. 229). A pairwise independent t-test with Holm-Bonferroni-adjusted p-values was run between all possible pairs of codon impact vectors shown in the table. The reported p-values in the table are the bootstrapped average of 1000 randomly drawn phage-host codon impact values, where codon impact represents the impact of each codon on the average phage-host codon mismatch. There is a significant difference between HP1 and *Pseudoalteromonas* sp strain 13-15, HS2 and *Pseudoalteromonas* sp strain 13-15, HP1 and Siphoviridae, and HP1 and all phages, denoted by the asterisks.

|  | PSA-HP1 | PSA-HS2 | Myoviridae | Siphoviridae | Podoviridae | All phages |
| --- | --- | --- | --- | --- | --- | --- |
| PSA-HS2 | 0.0002 ** |  |  |  |  |  |
| Myoviridae | 0.1785 | 0.2645 |  |  |  |  |
| Siphoviridae | 0.0075 ** | 0.9873 | 0.5390 |  |  |  |
| Podoviridae | 0.1036 | 0.3818 | 0.9141 | 0.6563 |  |  |
| All phages | 0.0330 * | 0.7920 | 0.7737 | 0.9139 | 0.8502 |  |

**Supplementary Methods:**

**Proteomics data quality assessment**

Given the few sample quantity and proteome quantity, a quality analysis was performed (Fig. S11-S13). The proteome samples that didn’t meet the expected thresholds of at least three of these metrics were excluded from downstream analyses (Fig. S11-S13). In total, only three samples were excluded: Con_T010_P_R2, HP_T010_P_R2, and HS_T010_P_R1. The first metric is the richness (number of distinct proteins) per sample, with the expectation that the within-replicates richness doesn’t deviate more than ±10% of the average richness of the three replicates. Richness was determined using the “specnumber” function of the package “vegan” in R. The second metric is the distance between samples in a Principal Coordinate analysis conducted on a Bray-Curtis dissimilarity matrix of the proteomes, with the expectation that all samples would fall within the 95% confidence interval for the same growth conditions upon challenging these proteomes with a different decoy proteome dataset generated from different growth conditions. The Bray-Curtis dissimilarity matrix was calculated using the function “vegdist” with the method “bray”, while the ordination was conducted using the function “capscale” with no constraints. The 95% confidence intervals were determined using the standard deviation method in the function “ordiellipse”; all of the three functions are part of the package “vegan” in R. The third metric is the module membership after hierarchical clustering followed by Dynamic Tree cutting (a top-down algorithm) or Dynamic Hybrid tree cutting (a bottom-up algorithm)(3), with the expectation that no sample should cluster with the decoy proteome dataset or emerge as a sole member of a module (singleton). Hierarchical clustering was conducted using the “pvclust” function with 10,000 bootstap iterations and using the average as the clustering method and the correlations for calculating the distances. Tree cutting was conducted using the “cutreeDynamic” function with a minimum cluster size of 3 and using the “tree” and “hybrid” methods; both functions are part of the package “pvclust” in R. The last metric is the pairwise correlations between the replicates of the same treatment and time point, with the expectation that the correlations should be as close as possible to 1. The correlations were calculated using the Pearson’s correlation method and correlations that were <0.97 were considered for exclusion after consulting the results from the other metrics. The in-house script used for the analyses described can be found in Cyverse (<http://datacommons.cyverse.org/browse/iplant/home/shared/iVirus/Pseudoalteromonas_Omics>).

**Supplementary References:**

1. Carver T, Harris SR, Berriman M, Parkhill J, McQuillan JA. Artemis: an integrated platform for visualization and analysis of high-throughput sequence-based experimental data. *Bioinformatics*. 2012;**28**:464–9.

2. Doron S, Fedida A, Hernndez-Prieto MA, Sabehi G, Karunker I, Stazic D, et al. Transcriptome dynamics of a broad host-range cyanophage and its hosts. *ISME J*. 2016;**10**:1437–55.

3. Langfelder P, Zhang B, Horvath S. Defining clusters from a hierarchical cluster tree: the Dynamic Tree Cut package for R. *Bioinformatics*. 2007;**24**:719–20.
